# STIM–Orai signaling in neurons: integrating calcium dynamics and synaptic plasticity

**DOI:** 10.1186/s12964-026-03010-y

**Published:** 2026-07-02

**Authors:** Joanna Gruszczynska-Biegala

**Affiliations:** Molecular Biology Unit, Mossakowski Medical Research Institute, Polish Academy of Sciences, Warsaw, Poland

**Keywords:** STIM, Orai, Store-operated calcium entry (SOCE), Calcium channels, Calcium signaling, Synaptic plasticity, Dendritic spines, Neuron

## Abstract

Precise regulation of intracellular calcium signaling is essential for neuronal function and depends on tightly coordinated molecular mechanisms. Among them, endoplasmic reticulum–plasma membrane (ER–PM) junctions form dynamic signaling microdomains that support store-operated calcium entry (SOCE), lipid transfer, phosphoinositide signaling, and ion channel regulation. Within this context, stromal interaction molecules STIM1 and STIM2 act as ER calcium sensors with distinct activation properties. STIM1 is recruited during substantial ER calcium depletion and strong neuronal activity, whereas STIM2 responds to subtle calcium fluctuations and supports basal calcium homeostasis. Upon ER calcium depletion, STIM proteins undergo conformational rearrangement, oligomerization, and translocation to ER–PM junctions, where they engage Orai calcium channels to trigger calcium influx and restore ER calcium balance. Although originally characterized in non-excitable cells, the STIM–Orai signaling axis is now recognized as an important regulator of neuronal calcium dynamics. This system exhibits considerable molecular diversity, with multiple STIM and Orai isoforms and splice variants differing in activation thresholds, subcellular localization, and signaling properties. Consequently, neuronal SOCE is not a uniform calcium entry pathway but contributes to specialized functions across dendrites, dendritic spines, and presynaptic compartments. Beyond classical SOCE, STIM proteins also function as multifunctional organizers of ER architecture and ER–PM junctions, linking calcium homeostasis to dendritic spine morphology, receptor trafficking, neurotransmitter release, and synaptic plasticity. Despite significant progress in this field, a comprehensive comparative understanding of the isoform-specific roles of STIM and Orai proteins in neuronal compartments remains limited. This review summarizes current knowledge regarding the functions of STIM1, STIM2, and Orai1–3 in dendrites and synapses, emphasizing their distinct contributions to calcium homeostasis, local signaling microdomains, and activity-dependent versus homeostatic forms of synaptic plasticity. By integrating evidence from genetic, biochemical, and in vivo studies, this review further highlights region-specific and context-dependent effects, unresolved controversies regarding the physiological roles of STIM and Orai, and the consequences of their dysregulation for neuronal function.

## Abstract (plain language summary)

Our brains rely on carefully controlled calcium signals inside neurons to function properly. Calcium helps neurons communicate, adapt, and store information. One of the key mechanisms underlying this regulation in neurons is the STIM–Orai pathway. STIM proteins sense calcium levels inside a cell structure called the endoplasmic reticulum. When calcium levels drop, STIM proteins move to special contact points at the cell surface and activate Orai channels, allowing calcium to flow back in and restore balance.

Although first studied in non-neuronal cells, STIM and Orai proteins are now known to have specific and diverse roles in neurons. Different types of STIM and Orai proteins are found in specific parts of neurons, including branches (dendrites) and the connections between neurons (synapses). They not only control calcium levels but also help organize cell structures and coordinate neuronal responses to activity.

This review brings together current knowledge about how STIM and Orai proteins regulate calcium, support communication between neurons, and contribute to the brain’s ability to change and adapt - processes important for learning and information processing. It also highlights questions that scientists are still trying to answer about how this system works in neurons and what happens when it becomes dysregulated.

## Introduction

Changes in intracellular calcium generate signaling gradients that regulate diverse neuronal processes, ranging from gene transcription to synaptic communication and cell survival (Berridge 1998, Brini et al 2014). Many of these calcium-dependent signaling pathways converge on synaptic plasticity, understood as the ability of neuronal connections to undergo long-lasting changes in strength and structure and to serve as the cellular basis of learning and memory (Berridge 1998, Brini et al 2014, Cavazzini et al 2005, Mateos-Aparicio & Rodríguez-Moreno 2020). These adaptive changes manifest as activity-dependent long-term potentiation (LTP) or long-term depression (LTD) and are tightly regulated by precise calcium signaling within both pre- and postsynaptic compartments. A key role in these processes is played by dendritic spines, the primary postsynaptic sites of excitatory synapses, which exhibit diverse morphologies ranging from immature filopodia-like protrusions to thin, stubby, and mushroom-shaped spines (Bourne & Harris 2008, Kasai et al 2003). Among these, mushroom spines are the most stable and functionally mature structures, and their activity-dependent structural and functional modifications are critical for synaptic efficiency and long-term information storage (Bourne & Harris 2007, Hayashi & Majewska 2005). In contrast, thin spines, distinguished by their small heads and high structural plasticity, are highly dynamic and facilitates synaptic remodeling during learning and memory formation (Bourne & Harris 2007).

For many years, calcium entry into neurons through N-methyl-D-aspartate (NMDA) receptors and voltage-gated calcium channels (VGCCs) has been regarded as the principal mechanism regulating synaptic plasticity (Higley & Sabatini 2012). However, early studies demonstrated that synaptic activity also induces calcium release from endoplasmic reticulum (ER) stores, significantly amplifying dendritic calcium signals in hippocampal CA1 neurons, particularly in young developing cells (Alford et al 1993, Emptage et al 1999). This ER-derived calcium release contributes substantially to synaptically evoked calcium transients in dendritic spines, whereas calcium signals triggered by back-propagating action potentials are largely independent of intracellular stores under physiological conditions (Emptage et al 1999).

Subsequent pharmacological studies revealed that depletion of ER calcium stores via sarco/endoplasmic reticulum calcium ATPase (SERCA) inhibitors, as well as inhibition of calcium influx activated by store depletion, termed store-operated calcium entry (SOCE), markedly impairs dendritic calcium signaling, short-term facilitation, and LTP at Schaffer collateral–CA1 synapses in the hippocampus (Baba et al 2003, Emptage et al 2001). These findings indicate that calcium influx through store-operated channels (SOCs) following ER calcium depletion is essential for sustaining activity–dependent synaptic signaling. Later studies confirmed the functional importance of neuronal SOCE, demonstrating that its activation following ER calcium depletion depends on the translocation of the calcium-sensing stromal interaction molecule (STIM) proteins (STIM1 and STIM2), with STIM1 as the primary mediator, and their subsequent interaction with Orai calcium channels in the plasma membrane, particularly Orai1. This mechanism enables extracellular calcium entry into the cytoplasm, thereby contributing to restoration of neuronal ER calcium homeostasis (Blaustein & Golovina 2001, Putney 2003).

Importantly, neurons exhibit unique spatial and temporal requirements, suggesting that SOCE contributes to synapse-specific and activity-dependent calcium microdomains within dendrites and spines. Early evidence, largely based on pharmacological manipulation of SOCE, demonstrated its involvement in both LTP and LTD, underscoring its fundamental role in mechanisms underlying learning and memory (Baba et al 2003, González-Sánchez et al 2017). More recent studies indicate that STIM and Orai proteins actively shape these localized calcium responses through isoform-specific and compartment-dependent mechanisms discussed in later sections of this review.

These compartmentalized calcium signals are organized in part by endoplasmic reticulum-plasma membrane (ER–PM) junctions, specialized membrane contact sites that act as multifunctional signaling hubs in neurons. Beyond regulating calcium homeostasis, ER–PM junctions coordinate inter-organelle communication, phosphoinositide signaling, kinase activity, lipid exchange, cytoskeletal organization, and receptor distribution at synapses. They also regulate organelle positioning and dynamics, including mitochondrial and endosomal trafficking and fission (Chanaday & Kavalali 2022, Guillén-Samander & De Camilli 2023). Through these integrated functions, ER–PM junctions support synaptic organization and plasticity through mechanisms that extend beyond direct calcium influx (Guillén-Samander & De Camilli 2023). Consistent with this integrative role, a direct link between structural and functional synaptic plasticity has been demonstrated in landmark studies showing that activity-dependent enlargement of individual dendritic spines is tightly coupled to the strengthening of synaptic transmission (Matsuzaki et al 2004).

This convergence of structural remodeling and functional potentiation highlights the coordinated nature of synaptic plasticity at the level of individual synapses and supports the concept that dendritic spines can function as discrete units of information storage. In this context, the ER forms a continuous yet highly dynamic network that extends throughout neurons into dendrites and, in a subset of synapses, into dendritic spines and presynaptic terminals (Berridge 1998, Chanaday & Kavalali 2022, Perez-Alvarez et al 2020, Spacek & Harris 1997, Wu et al 2017). Notably, only a fraction (15–50%) of dendritic spines contain ER or specialized ER-derived structures such as the spine apparatus, which are associated with enhanced synaptic stability and plasticity (Hering & Sheng 2001, Ng et al 2014, Perez-Alvarez et al 2020, Spacek & Harris 1997, Wu et al 2017). Synaptic activity has been shown to regulate ER dynamics by promoting ER recruitment into active spines and facilitating formation of ER–PM junctions. This activity-dependent redistribution enables STIM–Orai interactions and spatially restricted activation of SOCE at synaptic sites. Importantly, synaptic activity can rapidly remodel ER morphology and localization, thereby influencing local calcium signaling and contributing to compartmentalized synaptic responses (Ng et al 2014, Perez-Alvarez et al 2020).

Despite substantial progress, several important aspects of neuronal calcium homeostasis remain unresolved and are the subject of ongoing debate. These include the extent to which the ER functions primarily as a calcium source versus a buffering compartment, the relationship between SOCE and ER store refilling in neurons, the interplay between SOCE and calcium influx through VGCCs or NMDA receptors, and the impact of temperature on SOCE dynamics (Bell et al 2019, Pozzo-Miller et al 1997, Rae et al 2000). Although these issues are beyond the main scope of this review, they remain important open questions in the field.

Within these ER–PM signaling domains, STIM proteins, characterized by a single transmembrane domain, luminal EF-hand motifs that sense ER calcium levels, and cytoplasmic domains responsible for Orai channel activation, function as central regulators of cellular calcium homeostasis. These structural properties enable coupling of ER calcium depletion to Orai-mediated calcium entry, with important implications not only for neuronal signaling but also for broader physiological processes including, immune responses and muscle contraction (Emrich et al 2022).

Dysregulation of STIM-Orai signaling has been implicated in a broad spectrum of pathological conditions, including neurodegenerative diseases, cardiovascular disease, immune disorders, and cancer (Bollimuntha et al 2017, Chen et al 2019, Chen et al 2016, Cheng et al 2012, Feske et al 2010, Hogan et al 2010, Popugaeva et al 2017, Popugaeva et al 2015, Serwach & Gruszczynska-Biegala 2019, Wegierski & Kuznicki 2018, Zhang & Hu 2020, Zhang et al 2017). Notably, emerging evidence further indicates that aberrant STIM–Orai–dependent calcium signaling contributes to synaptic dysfunction and cognitive decline, highlighting the importance of this pathway for brain physiology and disease (Berna-Erro et al 2009, Popugaeva et al 2015, Steinbeck et al 2011, Sun et al 2014, Wu et al 2016, Wu et al 2011, Zhang et al 2016, Zhang et al 2015).

Two decades after the discovery of the STIM and Orai proteins, substantial progress has been made in elucidating their roles in non-neuronal contexts, particularly in the immune system. In contrast, the neuronal functions of SOCE components, especially in synaptic plasticity and memory, have only recently begun to be appreciated. This review focuses on STIM–Orai-mediated SOCE in neurons, with particular emphasis on the molecular mechanisms underlying calcium signaling in dendritic and synaptic compartments and their contributions to synaptic plasticity. Importantly, while several previous reviews have provided valuable insights into neuronal SOCE (Bouron 2023, Courjaret et al 2024, Korshunov & Prakriya 2025, Majewski & Kuznicki 2015, Segal & Korkotian 2016), they primarily addressed general aspects of neuronal SOCE or individual molecular components. The rapid expansion of the field now enables a more integrated mechanistic perspective on STIM–Orai signaling in dendritic and synaptic compartments.

The aim of this review is to integrate current knowledge and to advance our understanding of STIM and Orai proteins and the neuronal SOCE mechanism, with a particular focus on their structure, neuronal expression, and physiological roles in dendritic spines and synapses. In this context, this review outlines how STIM- and Orai-dependent SOCE shapes both local and global aspects of neuronal signaling, linking ER–PM junction dynamics to synaptic transmission, dendritic spine remodeling, and synaptic plasticity. Collectively, this framework highlights the role of STIM–Orai signaling in coordinating neuronal calcium homeostasis with information processing and cognitive function.

## STIM protein structure

In mammals, two main homologous STIM proteins, STIM1 and STIM2 have been identified. Both are type I single-transmembrane proteins located primarily in the membrane of the ER that sense calcium levels in the ER and regulate calcium influx into the cytoplasm when needed. Alternative splicing of both STIM homologues generates isoforms with distinct localization and functional properties, further diversifying their roles in calcium signaling (Darbellay et al 2011, Knapp et al 2022, Miederer et al 2015, Ramesh et al 2021, Rana et al 2015, Xie et al 2022). The STIM1 protein, consisting of 685 amino acids, is embedded either in the ER membrane (approximately 90%) or to a lesser extent in the plasma membrane (10%) (Jardin et al 2013, Keil et al 2010, Liou et al 2005, Moccia et al 2015, Roos et al 2005, Soboloff et al 2012, Spassova et al 2006). STIM2 is generally reported to comprise 833 amino acid residues (Roos et al 2005, Stathopulos et al 2009). However, it remains debated whether this represents the conventional isoform or whether STIM2 is translated from an alternative start site, resulting in a shorter protein (Williams et al 2001). Unlike STIM1, STIM2 is considered to be localized predominantly in the ER (Ercan et al 2012, Soboloff et al 2006). The structure of STIM proteins includes an N-terminal ER-luminal, a transmembrane (TM), and a C-terminal cytoplasmic domain (Moccia et al 2015, Soboloff et al 2012). In the ER luminal domain, STIM1 contains a recently identified zinc-binding region upstream of the canonical EF-hand (cEF), which contributes to STIM1 clustering and SOCE regulation (Alary et al 2025), followed by a cEF, which is responsible for calcium binding and serves as an ER calcium-sensor. Recent large-scale functional mapping of the STIM1 cEF-hand revealed that specific residues not only coordinate ER calcium sensing but also regulate STIM1 oligomerization and stability, providing mechanistic insights into how sequence variations can alter SOCE and contribute to human disease (Kamath & Matreyek 2025). This is followed by a non-canonical hidden EF-hand domain (hEF), which is unable to bind calcium but is essential for regulating the stability of the cEF domain, and then by the sterile alpha-motif (SAM) required for protein–protein interaction and oligomerization, which includes one N-glycosylation site in STIM2 and two in STIM1 (Johnson et al 2022, Kilch et al 2013, Stathopulos et al 2009). In a recent study using molecular dynamics simulations that mimic ER calcium depletion, F154, R155, K156, V178, and L179 were identified as specific residues in distinct regions of the SAM that are essential for stable STIM1 multimerization and thus SOCE activation (Sallinger et al 2024). The luminal STIM2 N-terminus shares > 80% homology with STIM1 (Stathopulos et al 2009), but subtle differences in the cEF-hand sequence give STIM2 a twofold lower affinity for calcium ions, making it more sensitive to small changes in ER calcium levels (Brandman et al 2007, Kraft 2015, Zheng et al 2011). While the SAM domains of STIM1 and STIM2 are similar, small differences in the STIM2 SAM domain increase its stability, reducing its rate of oligomerization during ER calcium depletion (Brandman et al 2007, Stathopulos et al 2009, Zheng et al 2008, Zheng et al 2011). A signal peptide (SP) at the N-terminus is cleaved during translation, with STIM2 having a longer SP than STIM1 does. The long 101-residue STIM2 SP seems to reduce ER localization, resulting in the accumulation of uncleaved cytosolic preSTIM2, which may interact with Orai1 at the plasma membrane to regulate basal calcium levels (Graham et al 2011).

The single transmembrane domain (TM; aa 214–233) anchors STIM proteins in the ER membrane and relays calcium-dependent conformational changes from EF–SAM unfolding to the cytosolic region. Upon ER calcium depletion, reorientation of the TM helices and coiled-coil formation propagate the activation signal to the C-terminal domain (Derler et al 2016, Hirve et al 2018, Ma et al 2015).

The STIM2 protein shares the same topology and more than 65% sequence similarity with STIM1, with the main distinction being in the C-terminal region beyond the coiled-coil domains. The C-terminal domain, which plays a crucial role in the translocation of STIM proteins to ER–PM junctions and in activating SOCE, contains three coiled-coil (CC) motifs (CC1 and CC2-CC3). Within this region, the calcium release-activated calcium (CRAC) activation domain (CAD; amino acids 342–448) encompasses the STIM1-Orai activating region (SOAR; amino acids 344–442). In addition, STIM proteins contain an inhibitory domain (ID), and a proline-serine-rich domain (P/S) in STIM1 or a proline-histidine-rich domain (P/H) in STIM2 (Butorac et al 2019, Derler et al 2016, Lewis 2020, Muik et al 2011, Muik et al 2008, Park et al 2009, Yuan et al 2009). Furthermore, mutations within the CC1–CC2 regions, including residues involved in the SOAR regulatory interface, have been shown to alter STIM2 activation and its coupling to Orai channels, highlighting the importance of this region in fine-tuning SOCE. In particular, residue L342 in the CC1 region is critical for maintaining intramolecular interactions between CC1 and CC3, which regulate STIM2 coupling to Orai1 and calcium entry (Subedi et al 2018). Compared with STIM1, residue E470 in the STIM2 CAD/SOAR domain has been recognized as a key factor responsible for inducing a more open conformation of the STIM2 CAD/SOAR region. This structural change enhances STIM2 protein trafficking to ER-PM junctions and facilitates low-level Orai activation even under resting conditions (Zheng et al 2018). Functionally, these structural differences collectively contribute to the slower activation kinetics of STIM2 compared to STIM1 and its reduced efficiency in coupling to and activating Orai channels (Wang et al 2014, Wang et al 2009). Other residues, including L485 in SOAR of STIM2 (corresponding to F394 in STIM1), have also been proposed to contribute to distinct Orai1 gating properties (Wang et al 2014). In STIM1, mutations at the equivalent F394 residue within the SOAR/CAD apex differentially affect channel activation by modulating CC1–SOAR interactions and stabilizing either a quiescent or active conformation, although their precise effects remain context-dependent, with different experimental approaches reporting divergent impacts on SOAR–CC1 interactions (Höglinger et al 2021, Shrestha et al 2022, Zhou et al 2023). Consistent with its specialized signaling properties, recent studies in neurons show that STIM2 is constitutively enriched at ER–PM junctions in dendrites, where it forms regularly spaced puncta that act as specialized dendritic ER–PM calcium signaling microdomains, thereby supporting its role in maintaining basal calcium homeostasis (Benedetti et al 2025). Despite its slower activation kinetics, the relatively small dynamic range of STIM2 activation may enable rapid responsiveness to subtle fluctuations in ER calcium levels, supporting sustained ER calcium homeostasis under physiological and pathological conditions, including neurodegenerative disorders such as Alzheimer's disease.

Additionally, the C-terminus contains both end-binding (EB)- interacting domain and a polybasic domain (PBD). The EB-binding domain allows STIM1 to associate with microtubule plus-EB proteins (e.g., EB1), thereby regulating its localization, trafficking along microtubules, and availability at ER–PM junctions (Grigoriev et al 2008, Sampieri et al 2009). Although EB1 binding is not essential for SOCE activation, it modulates its kinetics and amplitude of calcium entry by transiently trapping STIM1 at microtubule ends, delaying its translocation and preventing excessive or premature activation (Chang et al 2018, Chen et al 2019, Grigoriev et al 2008, Huang et al 2021, Wang et al 2018). This regulation is further influenced by ER calcium depletion and phosphorylation within the adjacent proline-serine–rich (P/S) region of STIM1 (Pozo-Guisado et al 2013, Pozo-Guisado & Martin-Romero 2013). In particular, phosphorylation of specific serine residues (e.g., Ser575, Ser608, and Ser621) promotes STIM1 dissociation from EB1 and facilitates its translocation to ER–PM junctions, whereas dephosphorylation restores STIM1–EB1 interaction. In neurons, STIM2 has been reported to associate with EB3-containing complexes via a related motif, suggesting a distinct but potentially analogous regulatory mechanism (Pchitskaya et al 2017). However, unlike STIM1, STIM2 does not exhibit microtubule plus-end tracking behavior.

The PBD, in turn, promotes STIM association with the plasma membrane through interactions with phosphoinositides such as PtdIns(4,5)P2 and PtdIns(3,4,5)P3, supporting ER–PM junction formation (Prakriya & Lewis 2015, Walsh et al 2009). Notably, STIM1 requires oligomerization for efficient membrane binding, whereas STIM2 exhibits higher intrinsic lipid affinity, which may underlie its preferential localization at junctions under resting conditions and its role in maintaining basal calcium levels. The PBD is not strictly required for Orai activation, indicating a primary role in spatial organization rather than channel gating (Bhardwaj et al 2013, Prakriya & Lewis 2015).

Both STIM1 and STIM2 contain multiple calcium/calmodulin (CaM)-binding sites within their C-terminal regions, including the SOAR domain. However, an important distinction lies in STIM2, where the CaM-binding site overlaps with the PBD. In STIM2, calcium/CaM binding can compete with phosphoinositide interactions, thereby modulating ER–PM junction stability and providing a calcium-dependent negative feedback on calcium entry (Bauer et al 2008, Bhardwaj et al 2013). In contrast, CaM binding to STIM1 primarily regulates Orai channel inactivation and the disassembly of STIM1–Orai complexes, rather than directly competing with membrane binding (Bauer et al 2008, Bhardwaj et al 2013, Li et al 2017). These differences suggest a more prominent role for CaM in fine-tuning STIM2 function, particularly in the regulation of basal calcium homeostasis.

## Structure of Orai

Orai is a membrane-spanning protein that constitutes the core component of CRAC channels, which are activated in response to the depletion of calcium stores in the ER (Feske et al 2006, Prakriya et al 2006, Vig et al 2006a, Vig et al 2006b). Subsequent studies demonstrated that Orai channels are functionally coupled to the ER calcium sensor STIM1, forming the minimal molecular unit of SOCE (Luik et al 2006). Thus, the primary function of Orai is to mediate the selective and tightly controlled influx of calcium ions from the extracellular space into the cytoplasm during SOCE.

Each Orai monomer consists of four transmembrane segments (designated TM1–TM4) spanning the plasma membrane, cytoplasmic N- and C-termini, and three short intra- and extracellular loops (Feske et al 2006, Prakriya et al 2006, Vig et al 2006b, Zhang et al 2006). A single monomer cannot conduct calcium ions. Instead, the functional channel is formed by the oligomerization of six monomers into a hexamer, which creates the central ion-conducting pore (Hou et al 2012, Thompson & Shuttleworth 2013). In this structure, the central calcium-conducting pore is formed by the first transmembrane segment (TM1) of each monomer, while TM2, TM3, and TM4 surround TM1, providing structural stability and transmitting conformational changes during activation (Derler et al 2013). Additionally, TM1–TM2 interactions are critical for channel gating, as mutations at these sites can constitutively open Orai1 (Prakriya & Lewis 2015, Yeung et al 2023). Different TM segments play distinct roles in channel activation and ion conduction. Although certain mutations or experimental manipulations can induce STIM1-independent channel opening, such activation has primarily been observed under non-physiological or engineered conditions and remains limited to specific experimental settings (Maltan et al 2023). Under physiological conditions, activation of Orai channels during SOCE remains strictly dependent on STIM proteins, which sense ER calcium depletion and couple it to channel gating. For example, photoactivation of specific residues within the Orai1 TM domains can induce CRAC-like calcium currents independently of STIM1, revealing intrinsic gating properties of the channel (Maltan et al 2023). However, the physiological relevance of this mechanism remains limited and is still under discussion.

The pore interior is lined with highly conserved amino acid residues, forming sequential functional zones responsible for channel selectivity and permeability (Prakriya et al 2006, Vig et al 2006a, Yamashita et al 2007, Yeromin et al 2006). The external vestibule at the channel entrance, composed of negatively charged aspartate residues (D110, D112, D114), forms an “electrostatic funnel” that attracts calcium ions toward the pore opening. This is followed by the selectivity filter, centered around the highly conserved glutamate residue E106, which is essential for the high selectivity of the channel toward calcium ions (Prakriya et al 2006, Yeromin et al 2006, Zhou et al 2010). Deeper within the pore is the hydrophobic cavity, which is composed of residues V102, F99, and L95 and contributes to ion flux stabilization and conformational modulation (Gudlur et al 2014, McNally et al 2012, Spassova et al 2008). The innermost region is the cytosolic basic zone and is composed of positively charged residues R91, K87, and R83, which may participate in pore closure and interactions with regulatory proteins.

The cytoplasmic N- and C-terminal domains of Orai play critical roles in interacting with STIM proteins (Derler et al 2013, Muik et al 2008, Palty & Isacoff 2016, Palty et al 2015, Park et al 2009, Yuan et al 2009). These domains contain motifs that enable direct binding to activated STIM1 or STIM2, which is essential for channel opening, although STIM1 generally exhibits stronger coupling and more efficient activation of Orai channels than STIM2. In the resting state, the Orai channel remains closed, with the calcium-conducting pore conformationally blocked. Upon depletion of ER calcium, STIM proteins oligomerize and translocate toward the plasma membrane, where they interact with Orai’s N- and C-terminal domains. This interaction induces a series of conformational changes that open the pore, allowing selective calcium influx from the extracellular space into the cytoplasm. In addition, the cytoplasmic C-terminus may contribute to fast calcium-dependent inactivation (FCDI), a rapid feedback mechanism limiting calcium entry, which is further regulated by a calcium-dependent inactivation domain within STIM1 that is functionally coupled to the Orai1 pore (Derler et al 2009, Mullins & Lewis 2016). Under some experimental conditions FCDI can occur even in the absence of STIM1, indicating that Orai1 possesses intrinsic structural mechanisms of this process (Prakriya & Lewis 2015, Yeung et al 2023). In contrast, slow calcium-dependent inactivation (SCDI) represents a distinct regulatory mechanism that likely requires additional regulatory factors, including STIM1 (Humer et al 2022, Jha et al 2013, Prakriya & Lewis 2015). Such a complex structural organization allows STIM-Orai complexes to respond precisely to changes in intracellular calcium levels and efficiently transmit calcium signals to both local and global signaling pathways in immune cells as well as neurons. From a physiological perspective, Orai1 is the best-studied and most important isoform in this family. Its role is particularly well documented in T lymphocytes, where its absence leads to severe combined immunodeficiency (SCID) (Feske et al 2006, Lacruz & Feske 2015, Picard et al 2009).

The other isoforms, Orai2 and Orai3 (Takahashi et al 2007), share a homologous structure with Orai1 but differ in their STIM sensitivity, calcium conduction efficiency, and pharmacological profiles (Hoth & Niemeyer 2013, Shuttleworth 2012). Despite this conservation, isoform-specific structural differences critically determine their interaction with STIM proteins and function at ER–PM junctions. All Orai isoforms share a conserved TM1 pore-forming region, including the selectivity filter residue E106 in Orai1, whereas the greatest variability is found in the N- and C-termini, and intracellular loops (Hoth & Niemeyer 2013, Tiffner & Derler 2021). The N-terminus of Orai1 contains polybasic and proline-rich motifs absent in Orai2 and Orai3, which strengthen STIM1 coupling and contribute to larger calcium currents (Fahrner et al 2009, Takahashi et al 2007, Tiffner & Derler 2021). In contrast, Orai2 and Orai3 display reduced current amplitudes and altered activation kinetics, consistent with weaker STIM coupling (Frischauf et al 2009, Frischauf et al 2011, Lis et al 2007). The C-terminus of Orai2 and Orai3 show enhanced coiled-coil propensity and distinct STIM-interacting properties compared to Orai1, potentially influencing STIM–Orai complex stability at ER–PM junctions (Frischauf et al 2009). Further differences in TM3 and intracellular loop2 affect gating and hydrophobic coupling, contributing to isoform-specific activation thresholds and partial STIM-independent activity, particularly in Orai3 under experimental conditions (Hoth & Niemeyer 2013, Srikanth et al 2010, Tiffner et al 2021). Orai2 and Orai3 also lack the N-glycosylation site present in Orai1 and show divergence in extracellular loop architecture, which may modulate ion selectivity and pharmacological sensitivity, especially in heteromeric channels (Gwack et al 2007, Shuttleworth 2012).

While Orai1 forms the principal subunit of neuronal CRAC channels, Orai2 and Orai3 can heteromerize with Orai1 to generate native CRAC channels with diverse stoichiometries or form functional channels independently (Lis et al 2007, Vaeth et al 2017, Zhang et al 2020). This structural diversity suggests that different Orai isoforms and their combinations can fine-tune calcium entry and downstream signaling. Incorporation of Orai3 into heteromeric channel complexes can alter redox sensitivity and modulate responsiveness to pharmacological agents such as 2-APB, thereby further diversifying CRAC channel function (Bogeski et al 2010, Emrich et al 2023, Kappel et al 2020). Notably, the extent and kinetics of calcium-dependent inactivation differ among Orai isoforms. FCDI is most pronounced in Orai3 and weakest in Orai1, while Orai2 displays intermediate properties (DeHaven et al 2007, Lis et al 2007, Yoast et al 2020). In contrast, SCDI appears to be more prominent in Orai1, whereas Orai2 and Orai3 show reduced or absent SCDI (DeHaven et al 2007, Humer et al 2022, Jha et al 2013, Lis et al 2007).

In neurons, the roles of Orai1 and Orai2 are becoming increasingly well understood. These channels, upon activation by STIM proteins, contribute not only to the replenishment of ER calcium stores but also participate in the activation of local calcium-dependent pathways in neurons, including calcium/calmodulin-dependent kinase II (CaMKII) and cAMP response element-binding protein (CREB), which are critical for long-term synaptic plasticity and memory trace formation (Garcia-Alvarez et al 2015b, Maneshi et al 2020, Shanmughapriya et al 2015, Zhang et al 2016).

## STIM and Orai proteins in the SOCE mechanism

The SOCE mechanism, also known as capacitative calcium entry, was first described in the 1980 s as a phenomenon in which calcium influx across the plasma membrane is activated in response to the depletion of calcium stores (Putney 1986). At the core of this pathway are STIM proteins, as well as calcium channels located in the plasma membrane. In the resting state, STIM proteins reside in the ER membrane, where they monitor luminal calcium concentration via their cEF-hand calcium-binding domains. This calcium-sensing step represents the initiating event of the SOCE (Liou et al 2005, Zhang et al 2005). The cytosolic CAD/SOAR region is held in an inactive conformation near the ER membrane through interactions with the CC1 domain (Derler et al 2013, Ma et al 2015, van Dorp et al 2021).

SOCE activation occurs in several stages (Lewis 2020). First, stimulation of metabotropic receptors (e.g., GPCRs or mGluRs) triggers the phospholipase C signaling pathway, leading to the production of inositol 1,4,5-trisphosphate (IP₃) (Berridge et al 2000). Ryanodine receptors (RyRs) are activated by increases in cytosolic calcium through a calcium-induced calcium release (CICR) mechanism, which can be triggered either by calcium influx or by IP₃ receptor-mediated calcium release (Fill & Copello 2002). In neurons, glutamate release activates postsynaptic NMDA and metabotropic receptors, leading to calcium influx and ER calcium release through both RyRs and IP₃Rs (Alford et al 1993, Baba et al 2003, Emptage et al 1999, Emptage et al 2001). A major source of this activity-dependent calcium entry is through NMDA receptors and VGCCs. In particular, calcium entry via L-type channels during glutamate-induced membrane depolarization can also initiate CICR from ER stores, thereby contributing to SOCE activation in an activity- and frequency-dependent manner (Dittmer et al 2017). The resulting ER calcium depletion is detected by STIM proteins (Berridge et al 2000, Hoth & Penner 1992). This leads to a conformational change in the STIM proteins that includes several structural transitions: (1) The EF-hand calcium-binding domain in the N-terminal region of STIM proteins detects ER calcium depletion and undergoes conformational unfolding and dimerization of the EF–SAM region, thereby initiating STIM activation (Hirve et al 2018, Schober et al 2019, Stathopulos et al 2009). (2) These luminal changes are transmitted across the membrane via TM domain, which acts as a structural relay between the ER lumen and the cytosolic region. Upon activation, the TM helices undergo rearrangement that brings them into closer apposition and promotes coiled-coil interactions, facilitating conformational coupling to the cytosolic domains (Fahrner et al 2014, Hirve et al 2018, Ma et al 2015, Muik et al 2009, Muik et al 2011). This process contributes to the release of the CC1α1 inhibitory clamp from the CAD/SOAR region (Fahrner et al 2014, Hirve et al 2018, Ma et al 2015). (3) As a result, the cytosolic C-terminal region undergoes a transition from a compact to an extended conformation, exposing coiled-coil domains and enabling STIM dimerization and higher-order oligomerization, which are essential for its signaling function (Fahrner et al 2014, Liou et al 2005, Ma et al 2015, Wu et al 2006, Zhang et al 2005, Zhou et al 2013). (4) Following these conformational changes, the CAD/SOAR region is exposed to enable binding with Orai1, and the C-terminal region extends toward the plasma membrane (Fahrner et al 2014, Ma et al 2015, Yang et al 2012, Yuan et al 2009, Zhou et al 2013). Thus, the oligomerized STIM proteins translocate to pre-existing, dynamic ER-PM junctions, where they are retained and enriched through interactions of the PBD with plasma membrane phosphoinositides (Cohen et al 2023, Walsh et al 2009, Yuan et al 2009). SOAR oligomerization enhances the efficiency of Orai1 activation by stabilizing STIM1–Orai1 interactions (Cohen et al 2023). Recently it was shown that each native SOCE complex typically contains only a few STIM1 dimers (around 7) and a single Orai1 channel, even after ER calcium depletion, suggesting that recruitment is modest and spatially restricted (Shen et al 2021). Then CAD/SOAR region of STIM subsequently interacts with the Orai1 C-terminal region, forming complexes at ER-PM junctions called puncta, which provide a platform for channel activation (Lewis 2020, Liao et al 2008, Liou et al 2007, Liou et al 2005, Ma et al 2015, Mercer et al 2006, Park et al 2009, Potier & Trebak 2008, Segal & Korkotian 2016, Wu et al 2014) but do not necessarily result in channel opening, as exemplified by inhibitory STIM2 variants such as STIM2β (Chung et al 2018). Structural and functional studies indicate that full activation of the Orai1 channel can be achieved by the engagement of only one of the two Orai1-activating sites within the dimeric SOAR molecule (Zhou et al 2015). (5) This interaction results in the opening of Orai channels and facilitates the flow of extracellular calcium into the cytoplasm (Luik et al 2008, Muik et al 2008). Calcium influx through Orai channels subsequently replenishes ER stores via SERCA pumps and restores intracellular calcium homeostasis. STIM proteins then revert to their inactive state, terminating the SOCE process.

Although STIM1–Orai1 represents the best-characterized SOCE configuration, alternative STIM–Orai combinations, including STIM2 with Orai2 or Orai3, also contribute significantly to SOCE in a region- and context-dependent manner (Hoth & Niemeyer 2013). Given the extensive structural and mechanistic insight available for STIM1–Orai1, this complex serves as a reference framework for understanding the functional diversity of other SOCE configurations. A more detailed discussion of alternative STIM–Orai combinations is presented later in the manuscript. Beyond this canonical configuration, STIM2 plays an important modulatory role in SOCE. Due to its lower calcium-binding affinity, STIM2 is activated by smaller decreases in ER calcium levels compared to STIM1. STIM2 is constitutively enriched at ER–PM junctions (Höglinger et al 2021, Subedi et al 2018, van Dorp et al 2021) and can facilitate STIM1 activation by promoting conformational remodeling of its C-terminal domain, thereby enhancing STIM1–Orai1 coupling even at relatively high ER calcium levels (Subedi et al 2018). Thus, STIM2 functions as a regulator that tunes the sensitivity and fidelity of SOCE signaling under near-resting conditions.

In non-excitable cells, such as lymphocytes, SOCE is the main pathway for calcium influx from outside the cell (Blaustein & Golovina 2001, Feske et al 2006, Parekh & Putney 2005, Prakriya et al 2006, Roos et al 2005). It was long believed that SOCE did not occur in neurons because of their easy and unlimited access to extracellular calcium through VGCCs and receptor-regulated (ROCCs) channels. However, accumulating evidence highlights the increasingly significant role of SOCE in excitable cells, such as smooth muscle (Emrich et al 2022, Nguyen et al 2013) and neurons across various regions of the central nervous system (Baba et al 2003, Bouron 2000, Bouron 2023, Brini et al 2014, Emptage et al 2001, Gruszczynska-Biegala et al 2011, Moccia et al 2015, Samtleben et al 2015, Serwach & Gruszczynska-Biegala 2019). In addition, functional SOCE pathways have been detected in human and mouse neural stem/progenitor cells (NSCs/NPCs) (Domenichini et al 2018, Gopurappilly et al 2018, Latoszek & Czeredys 2021, Li et al 2012, Somasundaram et al 2014, Tonelli et al 2012, Toth et al 2016), differentiated neurons from NPCs (Shin et al 2010), patient-specific induced pluripotent stem cell (iPSC)-derived neurons (Rizo et al 2022, Vigont et al 2021), mouse and human embryonic stem cells (ESCs) (Hao et al 2014, Huang et al 2017), and differentiated neurons from mouse and human ESCs (Gopurappilly et al 2019, Hao et al 2014).

Moreover, shortly after the discovery of STIM proteins as calcium sensors, the SOCE mechanism based on STIM proteins was reported in cortical neurons (Gruszczynska-Biegala et al 2011, Keil et al 2010, Klejman et al 2009, Ng et al 2011, Steinbeck et al 2011), cerebellar Purkinje neurons (Hartmann et al 2014), hippocampal neurons (Berna-Erro et al 2009, Park et al 2010), spinal ganglion neurons (Gemes et al 2011) and Drosophila neurons (Venkiteswaran & Hasan 2009).

Studies on rat cortical neurons indicate that STIM1 and STIM2 perform distinct yet complementary functions in the regulation of SOCE (Berna-Erro et al 2009, Gruszczynska-Biegala & Kuznicki 2013, Gruszczynska-Biegala et al 2011, Keil et al 2010, Klejman et al 2009). STIM1 has a relatively low affinity for calcium and preferentially activates Orai1 only after substantial or complete ER calcium depletion, making it the primary “emergency” activator responsible for the rapid replenishment of calcium in the ER (Bird et al 2009, Zheng et al 2008, Zhou et al 2009). In contrast, STIM2 is more sensitive to subtle fluctuations in calcium concentration, enabling it to finely tune resting cytoplasmic calcium levels and sustain modest, constitutive calcium influx even when ER stores are only partially depleted (Brandman et al 2007, Gruszczynska-Biegala et al 2011, Thiel et al 2013).

Under experimental conditions, the STIM–Orai interaction is also modulated by the extracellular state and pharmacological modulators. Agents such as membrane-permeable calcium chelators (EGTA, BAPTA-AM) or the selective SERCA pump inhibitor thapsigargin produce distinct puncta formation profiles and channel activation dynamics. In neurons, thapsigargin increases the number of puncta containing overexpressed yellow fluorescent protein (YFP)-tagged STIM1 and Orai1, but not those containing YFP-STIM2 and Orai1 (Gruszczynska-Biegala et al 2011, Klejman et al 2009). Notably, in wild-type rat cortical neurons, the association of STIM2 with Orai1 is enhanced in the presence of BAPTA-AM and under low-calcium conditions, as demonstrated by an in situ proximity ligation assay (PLA) and co-immunoprecipitation experiments. Upon thapsigargin-induced SOCE activation, the highest density of PLA signals is observed for STIM1 and Orai1, indicating the predominant formation of STIM1-Orai1 complexes (Gruszczynska-Biegala & Kuznicki 2013). The apparent differences between BAPTA-AM and thapsigargin likely reflect distinct activation thresholds and calcium sensitivities of STIM1 and STIM2, with STIM2 responding more readily to subtle reductions in calcium homeostasis that are less effectively captured under maximal store-depletion conditions induced by thapsigargin. In addition, methodological factors, including the use of overexpression systems, may bias the detection of STIM1–Orai1 puncta and underestimate STIM2 contributions under physiological conditions. Furthermore, ER calcium depletion by thapsigargin triggers punctate colocalization of STIM1 and Orai in neuronal growth cones, which may contribute to the lateral turning responses during growth cone motility (Mitchell et al 2012). Collectively, these findings suggest a differential engagement of STIM1 and STIM2 with Orai channels depending on the extent and mode of calcium depletion. The coordinated actions of STIM1 and STIM2 with Orai1 therefore provide neurons with both the capacity to respond rapidly to acute ER calcium depletion and the ability to maintain calcium homeostasis under resting conditions.

However, in the mouse nervous system, SOCE mechanisms differ across brain regions. In cortical and hippocampal neurons, Orai2 in combination with STIM2 appears to preferentially contribute to calcium entry, whereas in cerebellar neurons STIM1-dependent Orai2-mediated SOCE has been reported to be more prominent (Berna-Erro et al 2009, Hartmann et al 2014, Sun et al 2014). In dorsal root ganglion sensory neurons, both STIM1 and STIM2 contribute to SOCE, with Orai1 and Orai3 forming functional heteromultimeric channels to support calcium influx (Wei et al 2017). Importantly, beyond regional differences in STIM/Orai composition, post-translational modifications of these proteins may further shape their structure, localization, and functional properties, thereby adding an additional layer of regulation to SOCE mechanisms in the nervous system (Johnson et al 2022). Despite these advances, several methodological considerations should be taken into account when interpreting these findings. It is important to note that many of these conclusions are derived from studies employing overexpression systems, fluorescently tagged constructs, mutagenesis approaches, and heterologous expression models (e.g., HEK293 cells), which may not fully reflect endogenous protein behavior. While such approaches have been instrumental in dissecting STIM–Orai structure–function relationships, they can alter protein stoichiometry, localization, and clustering dynamics, potentially exaggerating puncta formation or interaction strength. In addition, antibody-based methods and proximity assays may be influenced by antibody specificity and epitope accessibility, and genetic validation using knockout or knockdown models has not been consistently applied across all studies. Notably, only a subset of studies has employed endogenous systems, including primary neurons or genetic loss-of-function models, which are critical for validating physiological relevance. Therefore, although these data collectively support region-specific and stimulus-dependent STIM–Orai coupling, the precise composition and dynamics of native SOCE complexes in neurons remain incompletely defined.

In addition, measurements of SOCE in neurons, particularly within dendritic spines, are subject to important methodological constraints. Many studies rely on genetically encoded calcium indicators (GECIs) such as GCaMP or chemical dyes, which can differ in sensitivity, dynamic range, and buffering capacity, potentially affecting the amplitude and kinetics of detected calcium signals. These limitations are especially relevant in dendritic spines, where small volumes and restricted diffusion can lead to signal compartmentalization and complicate quantitative comparisons with somatic responses. Furthermore, overexpression approaches may influence endogenous calcium dynamics and affect the interpretation of neuronal SOCE measurements. Differences between experimental systems, including cultured neurons, acute slices, and in vivo models, as well as genetic knockout versus overexpression approaches, should therefore be carefully considered when interpreting reported SOCE properties in neuronal compartments.

## Expression of SOCE proteins in the nervous system

The expression of SOCE-related proteins is widespread in the nervous system but displays pronounced isoform-, region- and species-specific patterns. STIM1 and STIM2 are widely expressed in rodent and human brains at both the mRNA and protein levels, although their relative abundance varies across brain structures (Courjaret et al 2024, Korshunov & Prakriya 2025, Moccia et al 2015, Serwach & Gruszczynska-Biegala 2020).

STIM1 is widely expressed in major brain regions, including cortex, hippocampus, cerebellum, thalamus, hypothalamus, striatum, dorsal root ganglion (DRG), and spinal cord (Gemes et al 2011, Gruszczynska-Biegala et al 2011, Hartmann et al 2014, Kikuta et al 2019, Klejman et al 2009, Ramesh et al 2021, Skibinska-Kijek et al 2009, Steinbeck et al 2011, Wei et al 2017, Xia et al 2014).

At the cellular level, STIM1 is primarily localized to rodent neuronal somata and dendrites of pyramidal neurons of the cortex and hippocampus as well as cerebellar Purkinje neurons, with evidence of presynaptic enrichment in specific regions such as the barrel cortex (Gross et al 2007, Hartmann et al 2014, Klejman et al 2009, Skibinska-Kijek et al 2009, Steinbeck et al 2011). STIM1B, a neuron-specific alternatively spliced variant of STIM1 is a shorter isoform compared to canonical STIM1 (Ramesh et al 2021). It can be recruited to nerve terminals due to exon 13B insertion, which introduces a peptide regulatory motif that alters C-terminal interactions and subcellular targeting, thereby influencing coupling efficiency to Orai channels. Functionally, STIM1B displays slower clustering at ER–PM junctions and reduced calcium-dependent inactivation, which may support sustained calcium entry (Ramesh et al 2021).

STIM2 expression is generally higher than STIM1 in rodent hippocampus, suggesting that STIM2 may represent a dominant isoform in these structures (Berna-Erro et al 2009, Gruszczynska-Biegala et al 2011, Kraft 2015, Lein et al 2007, Sun et al 2014, Xia et al 2014). Unlike in rodents, human brain tissue exhibits relatively higher STIM1 expression than STIM2 particularly in the cortex and hippocampus (Courjaret et al 2024, Steinbeck et al 2011), indicating species-specific regulatory differences. This pattern is also reflected in rodent neurons at the cellular level, with *Stim2* gene expression being approximately twice as high as that of *Stim1* in the cortical, hippocampal, as well as DRG neurons (Berna-Erro et al 2009, Chauvet et al 2016, Chen et al 2021, Gruszczynska-Biegala et al 2011, Klejman et al 2009, Moccia et al 2015, Skibinska-Kijek et al 2009, Wei et al 2017, Xia et al 2014). The expression of STIM proteins is cell-type specific, as STIM1 is predominantly present in freshly isolated astrocytes and STIM2 predominates in neurons, indicating distinct physiological roles (Gao et al 2016, Gruszczynska-Biegala et al 2011, Steinbeck et al 2011).

Orai isoforms are broadly expressed across the nervous systems of rats, humans, and cynomolgus monkeys, with expression detected throughout the brain, spinal cord, and root ganglia (Guzman et al 2014). All three Orai isoforms (Orai1-3) are expressed in the central nervous system, but with distinct expression profiles. Orai1 is consistently found at relatively low abundance compared with Orai2 and Orai3 (Berna-Erro et al 2009, Courjaret et al 2024, Gross et al 2007, Gwack et al 2007, Takahashi et al 2007, Wei et al 2017, Xia et al 2014, Zhang & Hu 2020), yet its functional importance is supported by genetic studies demonstrating profound alterations in neuronal activity, activity-dependent spine morphogenesis, and LTP following Orai1 manipulation (Dou et al 2018, Maneshi et al 2020, Tshuva et al 2017). This observations suggest that the physiological significance of Orai1 may exceed what would be anticipated based solely on its transcript abundance. Although pharmacological inhibition studies also support a role for Orai channels in these processes, currently available inhibitors do not selectively distinguish between Orai isoforms (Prakriya & Lewis 2015).

Despite lower transcript abundance, moderate to strong expression of Orai1 is detected in multiple brain regions, including cortex, hippocampus, cerebellum, and amygdala using complementary approaches such as immunohistochemistry, transcriptomics, proteomics, and western blotting (Czeredys et al 2013, Guzman et al 2014, Linde et al 2013, Maciąg et al 2019, Mitchell et al 2012, Secondo et al 2019). The Orai1 protein has also been detected in vitro in cultured cortical and hippocampal neurons, and dorsal horn neurons (Gruszczynska-Biegala & Kuznicki 2013, Gruszczynska-Biegala et al 2011, Klejman et al 2009, Korkotian et al 2014, Xia et al 2014). Functional studies using siRNA-mediated knockdown further confirmed Orai1 and Orai3 as components of SOCE in DRG neurons (Gross et al 2007, Wei et al 2017). Orai3 expression has also been reported in murine cerebellar cortex and human brain (Gwack et al 2007, Sjöstedt et al 2020). In contrast, Orai2 shows robust expression in the mouse brain, with particularly high levels in the hippocampus (dentate gyrus and CA1/CA3 subfields), cerebellum, cortex, and DRGs (Berna-Erro et al 2009, Chauvet et al 2016, Chen-Engerer et al 2019, Courjaret et al 2024, Gross et al 2007, Hartmann et al 2014, Ramesh et al 2021, Stegner et al 2019, Wei et al 2017), but appears less abundant in human brain (Gwack et al 2007, Lein et al 2007, Sjöstedt et al 2020).

## STIM and Orai proteins in the regulation of neuronal excitability, synaptic transmission, plasticity, and cognitive processes

The STIM1, STIM2, and Orai1–3 proteins play distinct yet complementary roles at the synapse (Table 1), which are discussed in detail below. This table highlights the distribution, activation patterns, molecular interactions, and physiological roles of STIM and Orai proteins across the dendritic and synaptic compartments. Each isoform contributes uniquely to calcium dynamics, the regulation of various receptors, and structural stability, with implications for both rapid and long-term neuronal adaptations.

**Table 1:** Features and functions of STIM and Orai isoforms in synaptic and dendritic compartments

Feature/Function in synaptic and dendritic compartments	STIM1	STIM2	Orai1	Orai2	Orai3
Localization	Dendrites; ER–PM junctions; dendritic spines (transient, debated), limited or indirect presence in presynaptic compartments (Hartmann et al 2014; Korkotian et al 2014; Ng et al 2011; Segal & Korkotian 2014; Chhikara et al 2025; de Juan-Sanz et al 2017)	Dendritic shafts; enriched in mature mushroom-type spines; presynaptic terminals; ER–PM junctions (constitutive, pre-clustered) (Pchitskaya et al 2017; Popugaeva et al 2015; Sun et al 2014; Zhang et al 2016; Chhikara et al 2025; Serwach et al 2023; Kushnireva et al. 2021; Chanaday et al 2021)	Dendrites; dendritic spines; enriched in immature hippocampal spines; presynaptic compartments; (Maneshi et al 2020; Korkotian et al 2017; Basnayake et al 2021; Chanaday et al 2021; Tshuva et al 2017; Gruszczynska-Biegala & Kuznicki 2013)	Dendritic shafts and spines; variable enrichment across brain regions (hippocampus, striatum) (Zhang et al 2016; Zhang & Hu 2020; Korkotian et al 2017, Wu et al 2018)	Mainly peripheral neurons (DRG) and MSNs; synaptic localization remains unclear (Wei et al 2017; Wu et al 2018)
Activation conditions	Activated during strong synaptic stimulation, ER Ca2⁺ depletion, and intense glutamatergic activity (mGluR and NMDAR-dependent) (Ng et al 2011*; *Hartmann et al 2014*; *Dittmer et al 2017*; *Chhikara et al 2025)	Activated by small ER Ca2⁺ fluctuations; operates under resting conditions and tonic NMDA receptor activity (Sun et al 2014*; *Zhang et al 2016*; *Chhikara et al 2025*; * Dickson 2025)	Activated mainly by STIM1 following strong ER Ca^2^⁺ depletion and high synaptic activity; engaged during glutamatergic/NMDAR signaling (Korkotian et al 2014*; * Segal & Korkotian 2014*; *Maneshi et al 2020)	Activated by STIM1/STIM2 (mainly STIM2) under basal/low-amplitude ER Ca^2^⁺ fluctuations and mGluR/IP3R -dependent signaling (Zhang et al 2016*; *Chen-Engerer et al 2019*; *Lalonde et al 2014)	Activated during SOCE in DRG neurons; may function together with Orai1 under conditions of elevated excitability (Wei et al 2017)
Main function	Coordinates SOCE, rapid ER remodeling and formation of ER–PM junctions; supports activity-dependent coupling between ER Ca^2^⁺ release and plasma membrane Ca^2^⁺ entry (Dittmer et al 2017*;* Dittmer & Dell’Acqua 2024*; *Chhikara et al 2025*; * Dickson 2025)	Maintains basal SOCE and stabilizes ER–PM junctions; acts as a scaffold for persistent ER–PM contacts; supports ER penetration into spines and spine apparatus integrity; supports constitutive coupling between ER Ca2⁺ levels and low-level plasma membrane Ca2⁺ entry (Chhikara et al 2025*; *Serwach et al 2023*; *Kushnireva et al 2020*; *Pchitskaya et al 2017)	Mediates rapid activity-dependent SOCE; amplifies NMDA receptor-mediated Ca^2^⁺ signals; supports ER Ca^2^⁺ refilling and dynamic spine remodeling (Maneshi et al 2020*; *Korkotian et al 2017*; *Basnayake et al 2021*; * Korkotian & Segal 2017*; *Dou et al 2018*; *Tshuva et al 2017)	Mediates constitutive, low-amplitude SOCE; supports ER Ca^2^⁺ homeostasis and maintenance of synaptic signaling competence in mature spines under basal conditions (Zhang et al 2016*; *Lalonde et al 2014)	Contributes to SOCE and neuronal excitability in DRG neurons; auxiliary role in Ca^2^⁺ influx regulation (Wei et al 2017)
Protein interactions in spines	Orai1, cytoskeletal proteins (septins, actin), synaptopodin, EB proteins, STIM2 (Hartmann et al 2014*; *Korkotian et al 2014*; * Segal & Korkotian 2014*; *Grigoriev et al 2008*; *Pavez et al 2019*; * Dhanya & Hasan 2021a*; *Chhikara et al 2025)	Orai1, Orai2, TRPC6, EB3, actin, synaptopodin, AMPA and NMDA receptors, CaMKII (Sun et al 2014*; *Zhang et al 2016*; *Garcia-Alvarez et al 2015a*; *Pchitskaya et al 2017*; *Rakovskaya et al 2025*; *Serwach et al 2023*; * Gruszczynska-Biegala 2020, 2016)	STIM1, STIM2, SERCA (Korkotian et al 2014*; *Chanaday et al 2021*; * Gruszczynska-Biegala & Kuznicki 2013*; *Korkotian et al 2017*; * Segal & Korkotian 2014*; *Basnayake et al 2021)	STIM2 (dominant), TRPC6, STIM1 (interaction less prominent in mature spines), Orai1 (Zhang et al 2016*; *Chen-Engerer et al 2019*; *Lalonde et al 2014*; *Wu et al 2018)	Orai1 (heteromeric complexes in DRG); STIM coupling less defined in synapsis (Wei et al 2017)
Effect on postsynaptic receptors	Indirectly modulates Ca2⁺ influx (e.g., via NMDAR-dependent signaling); no direct regulation of AMPA or NMDA endocytosis (Garcia-Alvarez et al 2015a*; *Serwach et al 2023*; *Yap et al 2017*; *Gruszczynska-Biegala et al 2020)	Regulates NMDA receptor endocytosis and limits excessive Ca^2^⁺ influx; promotes AMPA receptor phosphorylation and trafficking (Garcia-Alvarez et al 2015a*; *Serwach et al 2023*; *Gruszczynska-Biegala et al 2020)	Supports GluA1 trafficking, NMDA receptor-dependent Ca^2^⁺ amplification, and CaMKII activation (Maneshi et al 2020*; *Lim et al 2018)	Indirectly regulates glutamatergic receptor complexes via sustained Ca^2^⁺ entry and PSD95/CaMKII-dependent signaling (Zhang et al 2016)	Indirect effects on receptor activity remain unclear
Functional role/significance	Supports mGluR-dependent LTD, intrinsic excitability, rapid spine remodeling, and stabilization of sustained synaptic plasticity (Majewski et al 2017*; *Hartmann et al 2014*; *Ryu et al 2017*; *Dittmer & Dell’Acqua 2024*; *González-Sánchez et al 2017)	Supports basal synaptic stability and long-term plasticity (LTP/LTD); stabilizes basal Ca^2+^ homeostasis in mature synapses; maintains spine structure and stability, receptor composition and neuronal survival (Yap et al 2017*; *Sun et al 2014*; *Zhang et al 2016*; *Kushnireva et al 2020)	Facilitates activity-dependent synaptic plasticity, LTP, rapid spine remodeling, synaptogenesis, presynaptic neurotransmitter release, and neuron–astrocyte communication (Maneshi et al 2020*; *Chanaday et al 2021*; *Korkotian et al 2017*; *Tshuva et al 2017*; *Lim et al 2018)	Stabilizes mature dendritic spines (but not in MSNs; region-specific role) and supports homeostatic plasticity through sustained low-level SOCE; contributes to maintenance of synaptic integrity (Zhang et al 2016*; *Lalonde et al 2014*; *Wu et al 2018)	Likely auxiliary regulator of SOCE and excitability in peripheral neurons; CNS role remains poorly defined; no detectable role in dendritic spine maintenance or synaptic structural remodeling in striatal MSNs (Wei et al 2017*; *Wu et al 2018)
Consequences of dysfunction	Impaired ER-dependent Ca2⁺ signaling, spine remodeling, and presynaptic remodeling; altered filopodia growth during development;	Increased excitotoxic Ca^2^⁺signaling; disrupted basal Ca^2^⁺ homeostasis; decreased pCaMKII/PSD95 levels; ER disorganization; loss of mushroom spines and spine apparatus; impaired long-term synaptic plasticity; altered receptor trafficking	Impaired activity-dependent Ca^2^⁺ signaling; reduced short-term plasticity and LTP; defective spine remodeling and synaptic stabilization; altered GluA1 trafficking Impaired	Disrupted spine SOCE in hippocampus; impaired homeostatic Ca^2^⁺ signaling; decreased PSD95/pCaMKII signaling; subtle synaptic dysfunction; reduced spine stability in hippocampal but not striatal neuron	Reduced SOCE and neuronal excitability in DRG neurons; no detectable effect on dendritic spine density or synaptic structure in striatal MSNs, suggesting limited involvement in central synaptic maintenance (Wei et al 2017*; *Wu et al 2018)
	deficits in motor learning, memory consolidation, and cognitive flexibility (Shim et al 2013*; *Hartmann et al 2014*; *Ryu et al 2017*; * Dhanya & Hasan 2021a,b*; *Majewski et al 2017*; *Garcia-Alvarez et al 2015b)	memory and cognitive deficits (Sun et al 2014*; *Zhang et al 2016*; *Yap et al 2017*; *Berna-Erro et al 2009*; *Serwach et al 2023*; * Gruszczynska-Biegala 2020)	hippocampus-dependent learning and memory with sex-dependent cognitive and behavioral alterations (Maneshi et al 2020*; *Korkotian et al 2017*; *Chanaday et al 2021*; *Maciąg et al 2019*; *Dou et al 2018*; *Tshuva et al 2017)	mild or context-dependent behavioral changes (Zhang et al 2016*; *Stegner et al 2019*; *Wu et al 2018)	
Main experimental evidence	Cultured hippocampal neurons, conditional KO, overexpression systems, imaging of ER dynamics, electrophysiology	KD/KO hippocampal neurons, synaptosomes, imaging, electrophysiology	Conditional KO mice, KD neurons, dominant-negative mutants, overexpression models, spine imaging, electrophysiology	KD/KO studies, RNAi silencing, synaptosomes, MSN models, electrophysiology	Primarily KD studies in DRG neurons and striatal MSN models
KO/in vivo validation	Yes (cKO and double KO studies, transgenic OE mice) (Ryu et al 2017*; *Hartmann et al 2014*; *Garcia-Alvarez et al 2015b*; * Dhanya & Hasan 2021a*; *Majewski et al 2017)	Yes (KO and cKO studies) (Berna-Erro et al 2009*; *Yap et al 2017*; *Sun et al 2014*; *Chanaday et al 2021)	Yes (KO and cKO studies, transgenic OE mice) (Dou et al 2018*; *Maneshi et al 2020*; *Maciąg et al 2019)	Partial (KO and cKO studies; behavioral KO evidence limited) (Stegner et al 2019*; *Chen-Engerer et al 2019)	Limited/unclear

*KO* knockout, *cKO* Conditional knockout, *KD* Knockdown, *OE* Overexpression

### STIM1

#### STIM1 in Purkinje neuron physiology

Although the presence of STIM1 has been demonstrated in the cell bodies of neurons in various brain structures, distinct dendritic signals have been observed mainly in Purkinje neurons, which are characterized by an extensive dendritic tree with thousands of spines. Their formation and plasticity depend both on synaptic activity and regulatory proteins, whereas calcium compartmentalization enables precise control of synaptic signals, which is crucial for cerebellar function and motor coordination (Klejman et al 2009, Masoli et al 2025, Skibinska-Kijek et al 2009). The particularly high immunosignal level in Purkinje neurons and their dendrites suggests a key role of STIM1 in regulating calcium homeostasis in these cells.

Studies by Hartmann et al. (2014) provided further evidence for the presence and significant role of STIM1 in the dendrites of Purkinje neurons. It was shown that STIM1 is concentrated in subsurface regions of the ER, remaining largely excluded from dendritic spines, which represent key compartments for metabotropic glutamate receptor (mGluR)1-IP_3_-dependent signaling (Hartmann et al 2014, Nomura et al 2025). STIM1 cooperates with IP_3_R1 and the SERCA2 pump, whereas RyRs function independently, indicating the existence of specialized ER subdomains responsible for calcium release and store refilling that locally shape synaptic plasticity.

Interestingly, STIM1 also modulates calcium responses induced by mGluR1 activation and has been functionally associated with TRPC3-dependent signaling underlying slow postsynaptic excitatory potentials (EPSPₛₗₒw) in Purkinje cells (Gui et al 2024, Hartmann et al 2014). However, whether this regulation reflects direct STIM1-mediated TRPC3 activation or indirect effects related to ER–PM junction organization and local membrane signaling remains unresolved. Moreover, modulation by nitric oxide (NO) inhibits this influx through S-nitrosylation of STIM1. In the absence of neuronal nitric oxide synthase (nNOS) or under reduced NO signaling, STIM1 undergoes excessive activation and clustering. This increases calcium influx, disturbs calcium homeostasis, and potentially weakens the integrity of dendritic spines (Gui et al 2024, Tellios et al 2020). These observations suggest that STIM1 integrates multiple signaling inputs (e.g. mGluR1 and NO) to maintain local calcium balance and structural stability in Purkinje neuron dendrites, which in turn shapes spine morphology.

#### STIM1 in neuronal development and cytoskeletal dynamics

In developing neurons, STIM1 exhibits high mobility and participates in dynamic cellular processes such as spine formation and filopodia growth (Keil et al 2010, Kushnireva et al 2020). Importantly, STIM1 activity is modulated by neuronal activity. Blockade of synaptic transmission (e.g., with tetrodotoxin) induces STIM1-dependent calcium transients and promotes the formation of immature filopodial protrusions (Kushnireva et al 2020). Consistently, STIM1 is preferentially associated with filopodia in developing neurons, whereas its contribution decreases with maturation as stable dendritic spines are formed and STIM2 becomes more prominent.

Rather than acting as a direct regulator of cytoskeletal proteins, STIM1 appears to support cytoskeletal remodeling indirectly through its influence on ER dynamics and local calcium microdomains. Since neuronal maturation involves cytoskeletal reorganization regulated by microtubule end-binding (EB) proteins, including EB1, which stabilizes microtubules, and EB3, which coordinates the remodeling of microtubules and actin filaments in dendritic spines (Jaworski et al 2009, Nakagawa et al 2000, Stepanova et al 2003, Wu et al 2017), it is plausible that STIM1 may indirectly interact with these proteins, potentially influencing ER tubule elongation and actin polymerization. Indeed, STIM1 has been implicated in dendrite and axon development, as well as in ER remodeling at presynaptic terminals, suggesting a potential role in coordinating calcium signaling during neuronal development and in mature synapses (de Juan-Sanz et al 2017, Mitchell et al 2012, Pavez et al 2019, Shim et al 2013). This mechanism is based on the ability of STIM1 to link the ER with dynamic microtubule plus ends through interaction with EB1 and, in some contexts, EB3, enabling coordinated cytoskeletal reorganization and local calcium influx within filopodia (Grigoriev et al 2008, Huang et al 2021, Pavez et al 2019).

In the developing Xenopus spinal cord, STIM1-dependent SOCE operates in neuronal growth cones and contributes to the generation of localized and oscillatory calcium signals within filopodia (Shim et al 2013). These spatiotemporal calcium dynamics are essential for attractive growth cone responses to netrin-1 and for proper axonal guidance. Disruption of STIM1 function abolishes filopodial calcium transients, impairs calcium-dependent guidance responses, and leads to defects in midline axon navigation in vivo (Shim et al 2013). These findings suggest that STIM1-dependent SOCE is a key regulator of calcium signaling underlying neuronal development and growth cone guidance, supporting a model in which STIM1 couples ER dynamics to localized signaling events essential for neuronal morphogenesis. Together with Orai1, it also participates in axon pathfinding and in the response of neuronal growth cones to brain-derived neurotrophic factor (BDNF) and semaphorin-3a (Mitchell et al 2012).

#### STIM1 in ER remodeling and ER–PM junction organization

In large mushroom-type dendritic spines stabilized by the spine apparatus, which depends on synaptopodin, STIM1 and Orai1 are recruited to postsynaptic regions where they support ER calcium release and SOCE (Deller et al 2003, Korkotian et al 2014, Segal & Korkotian 2014, Yap et al 2020). Synaptopodin is therefore thought to organize local calcium microdomains that are important for activity-dependent spine remodeling and long-term spine stability. Through this structural and signaling framework, synaptopodin facilitates glutamate-induced calcium release, supports long-term spine survival and spine head enlargement during LTP in CA1 hippocampal neurons and granule cells (Deller et al 2003, Korkotian et al 2014, Vlachos et al 2009, Yap et al 2020). Importantly, synaptopodin regulates the selective recruitment of STIM1 and Orai1 to dendritic spines, promoting STIM1 targeting to mushroom spines, a localization lost in synaptopodin knockout (SP KO) neurons without affecting dendritic STIM1 distribution (Korkotian et al 2014, Segal & Korkotian 2014). These data identify synaptopodin as a structural organizer of STIM1–Orai1 complexes, conferring spatial specificity to SOCE in mature synapses. Consistently, proteomic analysis of the synaptopodin-associated spine apparatus has identified STIM1 as one of the ER-enriched proteins within this compartment, together with key calcium-handling components such as IP_3_Rs and RyRs, supporting the view that STIM1 is an integral component of specialized spine ER signaling domains (BioID2-synaptopodin proteome) (Falahati et al 2022). These results raise the question of what determines the selective recruitment of STIM1 and Orai1 to specific spines and the spine apparatus during synaptic activity.

The structural organization of dendritic ER and ER–PM junctions provides the physical basis for the activity-dependent recruitment of STIM1 to postsynaptic signaling domains, thereby linking ER calcium dynamics to localized calcium entry and synaptic remodeling. In addition to synaptopodin-dependent targeting, STIM1 also contributes to the dynamic remodeling of ER–PM junctions (Maciąg et al 2024). It has been shown that STIM1 promotes the formation and enlargement of perisynaptic ER–PM junctions, with its activation and clustering driven primarily by strong NMDA receptor activity or ER calcium depletion, whereas α-amino-3-hydroxy-5-methyl-4-isoxazolepropionic acid (AMPA) receptors and Cav1.2 channels play a minor role in this process (Chhikara et al 2025, Dickson 2025). Under conditions of NMDA receptor overactivation, STIM1 levels decrease in cortical synaptosomes while remaining stable at the plasma membrane, suggesting the relocation of STIM1 from postsynaptic to extrasynaptic regions (Serwach et al 2023). This activity-dependent redistribution may represent an adaptive mechanism that prevents excessive local calcium accumulation and helps maintain the spatial balance of calcium signaling in neurons. Notably, under physiological stimulation, glutamate-induced calcium influx via NMDA receptors triggers ER calcium depletion and local STIM1 activation near dendritic spines, suggesting spatially restricted rather than large-scale redistribution (Dittmer et al 2017).

Recent studies further demonstrate that STIM1 not only regulates ER–PM junction formation but also controls activity-dependent ER remodeling within dendritic spines. Specifically, STIM1 is required for the expansion of ER content during synaptic stimulation, a process that accompanies spine enlargement and supports sustained synaptic signaling (Dittmer et al 2017). Importantly, this regulation appears to depend not only on calcium influx but also on the structural interaction between STIM1 and Orai1 at ER–PM junctions. Indeed, the formation of STIM1–Orai1 complexes has been shown to be essential for maintaining spine enlargement and long-lasting synaptic plasticity (late-phase LTP; L-sLTP), whereas the initial phase of spine growth remains unaffected (Dittmer & Dell'Acqua 2024). These findings highlight a scaffolding role of STIM1 in stabilizing ER remodeling and sustaining activity-dependent structural plasticity, independent of classical SOCE-mediated calcium entry.

#### STIM1 in hippocampal dendritic spines and ER-dependent calcium signaling

In contrast to Purkinje neurons, where STIM1 is largely restricted to dendritic ER domains, STIM1 in hippocampal neurons has also been detected within dendritic spines and showed to actively contribute to local calcium signaling (Korkotian et al 2014, Ng et al 2011, Segal & Korkotian 2014, Skibinska-Kijek et al 2009). The activation of type I mGluR receptors by dihydroxyphenylglycine (DHPG; an agonist of group I/5 mGluR) induces calcium release from the ER, which subsequently leads to STIM1 oligomerization and its translocation toward ER–PM junctions, where it activates Orai channels and promotes SOCE-mediated calcium influx in hippocampal dendrites (Ng et al 2011). However, more recent evidence obtained using knock-in mouse models and Cre-mediated knockout of *Stim1* in hippocampal neurons indicates that STIM1 is predominantly localized at dendritic ER–PM junctions and only transiently associates with dendritic spines. In these studies, STIM1 was largely excluded from stable localizations within postsynaptic spines and presynaptic boutons, suggesting that its contribution to synaptic function is dynamic and activity-dependent rather than constitutive (Chhikara et al 2025). Consistently, pharmacological activation with thapsigargin or DHPG reduces STIM1 mobility in dendrites in a PLC- and IP₃-dependent manner, further supporting activity-dependent recruitment of STIM1-mediated SOCE to postsynaptic signaling domains (Ng et al 2011).

STIM1-dependent calcium entry becomes particularly evident during repetitive synaptic stimulation (≥ 1 Hz), when concurrent activation of NMDA receptors and RyRs leads to ER calcium release, STIM1 activation, and localized modulation of calcium signaling near active synapses. Importantly, STIM1 does not directly regulate endocytosis of NMDA or AMPA receptors in dendrites, indicating that its primary effects on synaptic function are unlikely to depend on receptor trafficking mechanisms (Garcia-Alvarez et al 2015a, Serwach et al 2023, Yap et al 2017). Instead, STIM1 appears to modulate postsynaptic signaling indirectly through regulation of local calcium dynamics and ER–PM calcium coupling. Consistently, STIM1 silencing in cortical neurons enhances NMDA receptor-mediated calcium influx (Gruszczynska-Biegala et al 2020), likely reflecting altered calcium homeostasis rather than direct interaction with NMDA receptor subunits (Serwach et al 2023). These findings further support the view that STIM1 primarily regulates the amplitude and spatiotemporal organization of postsynaptic calcium signals. These events are accompanied by ER remodeling within dendritic spines, enlargement of dendritic spines, and activation of downstream signaling pathways, including NFAT-dependent transcription (Dittmer et al 2017). Importantly, in spines containing ER, STIM1 has been shown to associate with Orai1 channels (Dittmer & Dell'Acqua 2024, Segal & Korkotian 2014), supporting the formation of localized calcium signaling microdomains rather than acting as an isolated driver of calcium influx. Together, these findings suggest that ER-associated STIM1–Orai1 signaling complexes may contribute to structural and functional stabilization of mature dendritic spines. However, whether such complexes represent a specific molecular correlate of long-term memory-associated spines remains unclear and requires further experimental validation.

#### STIM1 in synaptic plasticity (LTP and LTD mechanisms)

These spine-localized calcium signaling mechanisms provide the cellular basis for activity-dependent synaptic plasticity, which is further manifested at the level of long-term potentiation and depression. Genetic studies have revealed that STIM1 does not act as a universal requirement for classical long-term synaptic plasticity. In mice with forebrain-specific conditional deletion of *Stim1* and *Stim2* (Oh-Hora et al 2008) enhanced LTP has been observed in hippocampal CA3–CA1 synapses, suggesting that STIM-dependent signaling may also act as a modulatory constraint on synaptic potentiation under certain conditions demonstrated increased LTP in excitatory transmission, especially in the CA3–CA1 pathway (Garcia-Alvarez et al 2015b).

Conversely, STIM1 overexpression in transgenic mouse neurons does not alter basal synaptic transmission, short-term plasticity, or hippocampal LTP, but selectively impairs mGluR-dependent LTD induced either electrically or by DHPG, without affecting NMDA receptor-LTD (Majewski et al 2017). This deficit does not result from presynaptic dysfunction and is only partially dependent on Orai-mediated SOCE, suggesting that excessive STIM1 expression disrupts finely tuned postsynaptic calcium signaling required for LTD expression (Majewski et al 2017). Investigating the molecular mechanisms underlying the partial calcium-independent effect of STIM1 on mGluR-dependent LTD in cortical neurons remains an important open question. Together, these findings indicate that STIM1 is not strictly required for LTP induction or maintenance, but can selectively regulate specific forms of synaptic depression, particularly mGluR-dependent LTD.

In Purkinje neurons, conditional deletion of *Stim1* reduces mGluR1-dependent synaptic potentials and alters calcium clearance and intrinsic neuronal excitability, while leaving classical long-term synaptic plasticity largely intact (Hartmann et al 2014, Ryu et al 2017). These findings further support the idea that STIM1 primarily regulates calcium dynamics and intrinsic excitability rather than directly controlling canonical LTP/LTD mechanisms in all neuronal types. In contrast, in cortical neurons, SOCE has been shown to be required for DHPG-induced mGluR-LTD, as pharmacological blockade of SOCE prevents LTD induction. Importantly, these experiments targeted SOCE channels rather than STIM proteins directly, indicating that STIM1–Orai-mediated calcium entry is a key upstream component of LTD signaling in this region (González-Sánchez et al 2017).

Recent evidence suggests that the interaction between STIM1 and Orai1 may contribute to the transition from short-term transient structural spine plasticity (early-phase LTP; E-sLTP) to more stable, consolidated long-term form (L-sLTP), suggesting a role in stabilizing activity-dependent changes associated with long-term potentiation (Dittmer & Dell'Acqua 2024). In spines lacking ER, stimulation-induced plasticity appears transient and weak, whereas recruitment of ER to such spines restores STIM1–Orai1-associated signaling and promotes more persistent structural remodeling. However, recent studies indicate that neuronal ER–PM junctions are structurally heterogeneous and may include distinct subpopulations associated with RyR- and Cav-dependent calcium signaling that are functionally separate from classical STIM–Orai-mediated SOCE domains (Benedetti et al 2025).

Overall, STIM1 contributes to the coordination of local dendritic calcium signaling and ER dynamics, where its coupling with Orai1 may influence whether plasticity remains transient or is consolidated into long-term changes associated with learning and memory (Dittmer & Dell'Acqua 2024). STIM1 functions in a context-dependent manner, integrating ER calcium dynamics, spine structural organization (including synaptopodin-dependent scaffolds), and receptor-specific signaling pathways rather than acting as a universal determinant of LTP or LTD. In this context, STIM1–Orai-mediated signaling regulates local calcium microdomains that selectively gate distinct forms of synaptic plasticity, particularly in mGluR-dependent pathways, in a region- and state-dependent manner (Aloni et al 2019, Deller et al 2003, Dittmer & Dell'Acqua 2024, Hartmann et al 2014, Korkotian et al 2014, Majewski et al 2017, Ryu et al 2017, Segal & Korkotian 2014, Vlachos et al 2009). The apparently divergent experimental results across systems reflect the multifunctional and context-sensitive nature of STIM1 signaling rather than a single, uniform role in either LTP or LTD expression.

#### STIM1 in synaptic excitability and presynaptic function

Beyond long-term synaptic plasticity, STIM1 also contributes to the regulation of baseline synaptic excitability at both pre- and postsynaptic sites (Chanaday et al 2021). Early studies demonstrated that depletion of ER calcium stores can trigger SOCE in presynaptic neurons and modulate spontaneous neurotransmitter release, although the specific contribution of STIM proteins was not directly addressed (Emptage et al 2001). These observations are further supported by studies in hippocampal neurons, where STIM1 deficiency leads to a reduced frequency and amplitude of mEPSCs, indicating its involvement in regulating neurotransmitter release probability and presynaptic activity (Chanaday et al 2021, de Juan-Sanz et al 2017).

Importantly, the neuronal splice variant STIM1B preferentially localizes to the presynaptic ER, where it counteracts synaptic depression during high-frequency stimulation. Due to its slower calcium-binding kinetics, STIM1B can transform classical synaptic depression into calcium- and Orai-dependent short-term synaptic enhancement (STE), promoting mobilization of the reserve pool of synaptic vesicles and sustaining neurotransmission during prolonged activity (Ramesh et al 2021). However, studies in Purkinje neurons indicate that STIM1 primarily regulates intrinsic excitability and calcium clearance (Hartmann et al 2014, Ryu et al 2017). Notably, STIM1-dependent regulation of excitability may also be age-dependent, as changes in ion channel expression have been observed in older but not younger knockout animals (Dhanya & Hasan 2021a). Importantly, although functional studies support a role for STIM1 in regulating presynaptic activity, recent high-resolution imaging data indicate that STIM proteins are largely excluded from presynaptic boutons and do not accumulate at synapses. Instead, they appear to transiently pass through axonal ER networks, suggesting that their effects on neurotransmitter release may be indirect and mediated by global ER dynamics rather than stable presynaptic localization (Chhikara et al 2025). Together, these findings suggest that STIM1 modulates synaptic excitability primarily through activity-dependent regulation of ER calcium dynamics and presynaptic function, although the precise mechanisms linking STIM1 signaling to neurotransmitter release remain to be fully defined.

#### Behavioral consequences of STIM1 dysregulation

At the systems level, alterations in STIM1 expression or function lead to consistent and measurable behavioral changes across multiple brain circuits.

In Purkinje neuron-specific knockout models, loss of *Stim1* impairs motor learning and locomotor performance, indicating a critical requirement for STIM1 in cerebellar-dependent motor control (Hartmann et al 2014). These deficits are associated with disrupted cerebellar information processing, as evidenced by impaired motor coordination and learning capacity. Conditional deletion of *Stim1* in mouse Purkinje neurons results in deficits in cerebellar memory consolidation, while leaving initial learning phases largely intact (Ryu et al 2017). Notably, learning-induced intrinsic plasticity is markedly impaired, suggesting that STIM1-dependent regulation of neuronal excitability is a critical component of memory storage mechanisms (Jang et al 2020). This dissociation suggests that STIM1 is particularly important for the stabilization rather than acquisition of motor memory, likely through its role in intrinsic plasticity of Purkinje cells. Consistent with this, long-term structural and functional alterations following *Stim1* loss further support its role in maintaining cerebellar circuit integrity (Dhanya & Hasan 2021a). In aged mice, Purkinje neuron-specific *Stim1* deletion leads to altered dendritic morphology, changes in gene expression, and abnormal climbing fiber innervation patterns, suggesting a role of STIM1 in maintaining the structural integrity of neuronal networks (Dhanya & Hasan 2021b). Remarkably, in these STIM1-deficient mice, partial or complete deletion of septin 7 (a GTPase that organizes the cytoskeleton and cellular functions) reversed the described changes, improving motor performance and synaptic connections between climbing fiber axons and dendrites of these neurons (Dhanya & Hasan 2021a). This indicates that STIM1-related dysfunction can be partially compensated by cytoskeletal reorganization mechanisms. It is therefore worth considering whether the interaction of STIM1 with cytoskeletal elements, such as septins, constitutes a potential compensatory mechanism in cerebellar calcium dysregulation.

In hippocampal and forebrain circuits, STIM1 also contributes to higher-order cognitive functions. Conditional forebrain deletion of *Stim1* and *Stim2* leads to impaired spatial memory and reduced anxiety-like behavior, whereas single *Stim1* deletion affects cognitive flexibility in reversal learning tasks (Garcia-Alvarez et al 2015b). In contrast, targeted optogenetic activation of STIM1 in the CA1 hippocampal region enhances contextual memory without affecting auditory memory, suggesting a selective role of STIM1 in hippocampus-dependent cognitive behavior (Kim et al 2020, Kyung et al 2015). These findings have been linked to learning-related behaviors, including spatial learning (Deller et al 2003), although such effects are indirect and mediated through synaptic plasticity mechanisms rather than representing a direct behavioral role of STIM1 signaling.

Gain-of-function studies further support a modulatory role of STIM1 in behavior. Transgenic mice that overexpressed neuronal STIM1 have reduced anxiety-like behavior and enhanced contextual learning without affecting locomotor activity (Majewski et al 2017), indicating that changes in STIM1 levels can bidirectionally regulate cognitive and affective behaviors.

Collectively, these findings demonstrate that STIM1 is not required for basic motor or sensory function but is essential for the fine regulation of motor learning, memory consolidation, cognitive flexibility, and affect-related behaviors. These observations support a view in which STIM1 acts as a central regulator of experience-dependent plasticity across multiple brain circuits, linking intracellular calcium dynamics to adaptive behavioral responses at the systems level.

### STIM2

#### STIM2 in dendritic spines and basal SOCE signaling

STIM2 in hippocampal neurons is preferentially enriched in mature “mushroom”-type dendritic spines, which represent stable excitatory synapses and play a key role in regulating local calcium signaling and synaptic plasticity (Pchitskaya et al 2017, Popugaeva et al 2015, Rakovskaya et al 2025, Sun et al 2014, Zhang et al 2016, Zhang et al 2015). Unlike STIM1, which is primarily activated during strong stimulation, STIM2 is partially localized in ER–PM junctions even under resting conditions, allowing it to detect small fluctuations in ER calcium levels and maintain constitutive, low-level SOCE. Following mild ER calcium depletion, STIM2 undergoes activity-dependent clustering and can be transiently recruited to spine heads, where it interacts with Orai channels (predominantly Orai2 or Orai1, depending on neuronal type), forming puncta that generate a sustained but low-amplitude calcium influx. This basal calcium entry contributes to the stabilization of spine size and supports postsynaptic receptor maintenance (Korkotian & Segal 2017, Segal & Korkotian 2014, Zhang et al 2016). STIM2–Orai1 complexes have also been detected in rat cortical neurons (21 DIV) using proximity ligation assay, primarily in cell bodies, although weak signals have also been observed in dendrites and probably in synapses (Gruszczynska-Biegala & Kuznicki 2013). Notably, STIM2 has also been detected in synaptosomes of cortical neurons, further supporting its synaptic localization beyond hippocampal systems (Serwach et al 2023).

#### STIM2–CaMKII pathway and structural spine stability

In striatal medium spiny neurons (MSNs), a reduction in STIM2 leads to partial loss of spines, suggesting that this protein may also perform an additional function independent of the classical SOCE mechanism in maintaining dendritic spine structure (Wu et al 2016). Pharmacological inhibition of neuronal SOCE similarly reduces MSN spine density both in vitro and in vivo, further supporting the role of STIM2-gated SOCE in structural spine stability. Interestingly, striatal neurons appear to be sensitive not only to reduced but also to excessive SOCE activity, as STIM2 overexpression also disrupts spine maintenance (Wu et al 2016). These observations indicate that precise regulation of basal calcium influx is required for proper spine homeostasis in the striatum and suggest that STIM2 may participate in dynamic ER remodeling, thereby indirectly affecting synaptic plasticity.

In hippocampal neurons, spine integrity critically depends on neuronal STIM2-dependent SOCE activity mediated by Orai channels, which maintains the phosphorylated form of CaMKII (pCaMKII), a key determinant of mature mushroom spine stability and synaptic integrity. STIM2 colocalizes with pCaMKII in mushroom spines, and the knockdown or knockout of STIM2 reduces both pCaMKII and PSD95 levels, leading to shrinkage and destabilization of mature mushroom spines, an increased proportion of immature thin spines, and consequently neuronal degeneration and loss of hippocampal integrity (Sun et al 2014, Zhang et al 2016). Conversely, STIM2 overexpression restores pCaMKII levels and spine density.

Importantly, conditional deletion of *Stim2* causes a dramatic reduction in synaptic SOCE activity in dendritic spines (~ 85%), whereas somatic SOCE is affected to a much lesser extent (~ 35%), indicating that STIM2 is the predominant regulator of synaptic SOCE in hippocampal neurons (Sun et al 2014). In vivo stereotaxic deletion of *Stim2* in the hippocampus produces comparable effects, further supporting the physiological relevance of this pathway (Sun et al 2014).

Notably, STIM1 cannot effectively compensate for STIM2 deficiency, most likely due to differences in calcium sensitivity and subcellular localization between these isoforms (Sun et al 2014). Consistently, STIM1 overexpression only modestly enhances synaptic SOCE, whereas STIM2 overexpression produces a markedly stronger increase in spine-associated calcium entry. However, unlike in striatal neurons, elevated STIM2 expression in hippocampal neurons does not substantially destabilize mature spines, indicating important region-specific differences in the regulation and functional consequences of SOCE signaling (Sun et al 2014, Wu et al 2016).

In hippocampal neurons, SOCE drives tonic synaptic CaMKII activity required for the maintenance of mature mushroom spines, whereas the downstream effectors of STIM2-dependent signaling in MSNs remain unclear (Sun et al 2014, Wu et al 2016). Given the high abundance of CaMKII in the striatum and its enrichment within MSN spines, it is possible that CaMKII also contributes to MSN spine maintenance, although this requires further investigation. Overall, the STIM2–SOC–CaMKII pathway appears essential for maintaining basal calcium homeostasis, mature synaptic structure, and neuronal survival. In contrast to STIM1, which primarily supports transient activity-dependent calcium signals, STIM2 functions predominantly as a homeostatic regulator that stabilizes synaptic structure under resting or mildly active conditions (Sun et al 2014, Zhang et al 2016). Consistent with this concept, cortical neurons exposed to ER stress undergo ATG7-dependent autophagic degradation of STIM2, which suppresses SOCE, reduces PSD95 and CaMKII levels, and ultimately promotes dendritic degeneration (Zhou et al 2019). These findings further support the idea that sustained STIM2-dependent calcium signaling is required for preservation of postsynaptic structural integrity under both physiological and stress-related conditions. Further studies are needed to determine how STIM2 maintains persistent CaMKII activation and how this pathway interacts with other kinase networks controlling dendritic spine architecture.

#### STIM2 in ER–PM junctions and scaffolding functions

Beyond its role in calcium signaling, STIM2 contributes structurally to the organization of ER–PM junctions. It is partially pre-clustered in dendrites and stabilized by tonic NMDA receptor activity, supporting the persistence of ER-PM junctions even under resting conditions (Benedetti et al 2025, Chhikara et al 2025, Dickson 2025). In contrast to STIM1, which is recruited during strong stimulation and primarily contributes to transient, activity-dependent ER–PM remodeling, STIM2 appears to be constitutively associated with perisynaptic ER-PM regions, where it supports stable ER–PM coupling and organization of local calcium signaling microdomains (Benedetti et al 2025, Chhikara et al 2025). Notably, approximately 82% of the periodically organized dendritic ER–PM puncta were found to contain STIM2. These STIM2-enriched signaling hubs colocalize with junctophilin-3, RyRs, and subsets of VGCCs, supporting coordinated calcium signaling within dendritic compartments (Benedetti et al 2025). Recent findings further indicate that neuronal ER–PM junctions form structurally heterogeneous calcium signaling microdomains, including RyR-associated domains that may operate independently from classical STIM–Orai SOCE sites (Benedetti et al 2025).

STIM2-dependent organization of these junctions relies on its interactions with cytoskeletal components, including microtubule end-binding proteins (e.g., EB3) and actin. These interactions regulate the distribution of ER tubules in spines and the formation of ER–PM junctions in dendrites (Serwach & Gruszczynska-Biegala 2020). Binding of STIM2 to EB3 is necessary for the formation of STIM2 clusters and maintenance of SOCE activity in dendrites. After ER calcium depletion, STIM2 can be dynamically recruited from the spine neck to the spine head, where it enhances local calcium signals that stabilize glutamatergic synapse function (Kushnireva et al 2020).

Functionally, STIM2-EB3 interactions facilitate structural remodeling of dendritic spines and promote the formation of mushroom-type spines in hippocampal neurons (Pchitskaya et al 2017). Consistently, STIM2 also regulates the distribution of synaptopodin, an actin-associated marker of the spine apparatus (Rakovskaya et al 2025). Increased STIM2 expression shifts synaptopodin clusters from dendrites into spines, facilitating the extension and stabilization of ER structures within dendritic spines and increasing the proportion of spines containing a spine apparatus. Conversely, disruption of STIM2 interaction with microtubules, for example through mutations in the EB3-binding site, impairs STIM2 recruitment and clustering in spines. This leads to ER redistribution toward the soma, reduced ER content in spines, decreased SOCE activity in hippocampal neurons, and consequent spine destabilization, including reduced size and number of spines containing a spine apparatus (Pchitskaya et al 2017, Rakovskaya et al 2025).

Together, these findings identify STIM2 as a key stabilizing scaffold for ER–PM junctions, linking cytoskeletal dynamics with ER positioning and the maintenance of structural substrates required for synaptic plasticity (Chhikara et al 2025). These observations also raise the possibility that STIM2-dependent ER–PM junctions may serve as morphological indicators of dendritic spines maturity and stability, although this hypothesis requires further experimental validation.

#### STIM2 in receptor regulation (NMDA and AMPA pathways)

STIM2 plays an important role in regulating glutamate receptor dynamics. Following short-term NMDA receptor overactivation, STIM2 silencing increases receptor surface expression, decreases receptor internalization from the synaptic plasma memrane, reduces NMDA receptor colocalization with endosomes, and enhances NMDA-induced currents, thereby inhibiting receptor endocytosis from the synaptic surface (Serwach et al 2023). This phenomenon is accompanied by an increase in STIM2 clustering, its interaction with GluN2A and GluN2B subunits of NMDA receptors (Chhikara et al 2025, Serwach et al 2023), and NMDA-induced calcium entry (Gruszczynska-Biegala et al 2020), suggesting that STIM2 normally acts to limit excessive calcium influx and protect neurons from excitotoxicity.

In addition, STIM2 regulates AMPA receptor function through both SOCE-dependent and SOCE-independent mechanisms. Under conditions of ER calcium depletion STIM2 facilitates calcium entry via AMPA receptors and forms complexes with these receptors (Gruszczynska-Biegala et al 2016). Consistently, pharmacological inhibition of SOCE reduces calcium entry via AMPA receptors, further supporting STIM2 involvement in this process. Independently of SOCE, STIM2 (but not STIM1) also promotes GluA1 phosphorylation via cAMP/protein kinase A (PKA) pathway at ER–PM junctions and enhances its trafficking to dendritic spines (Garcia-Alvarez et al 2015a). This results in increased GluA1 surface expression through coordinated regulation of exocytosis and endocytosis, thereby promoting dendritic spine maturation (Garcia-Alvarez et al 2015a, Garcia-Alvarez et al 2015b).

In addition to direct regulation of glutamate receptor trafficking, STIM2 also influences the lipid composition of synaptic membranes. It has been shown to control glutamate-induced cholesterol loss in synapses through the mobilization of the enzyme CYP46A1 to the plasma membrane (Sodero et al 2012). This process affects the amplitude of the depolarization-induced calcium response, highlighting the role for STIM2 in regulating neuronal membrane organization during glutamatergic excitation. Since cholesterol is a key component of synaptic membranes, essential for proper synaptic transmission and plasticity, STIM2-dependent regulation of its turnover may link calcium signaling with structural and functional remodeling of the synaptic membrane, particularly under conditions of excitatory or excitotoxic stress.

#### STIM2 in synaptic plasticity (LTP and LTD mechanisms)

Functional studies demonstrate that STIM2 is essential for long-term synaptic plasticity. Conditional knockout of *Stim2*, but not *Stim1*, disrupts both LTP and LTD in CA3–CA1 synapses and impairs activity-dependent AMPA receptor trafficking, including surface expression of GluA1 during LTP induction and its endocytosis under LTD conditions (Yap et al 2017). These findings indicate that STIM2 is required for bidirectional synaptic plasticity and proper regulation of AMPA receptor dynamics.

However, the reported role of STIM2 in hippocampal plasticity is not fully consistent across studies. In contrast to the impaired plasticity observed in STIM2-deficient neurons, combined deletion of *Stim2* and *Stim1* paradoxically enhances LTP and increases phosphorylation of GluA1, the transcription factor CREB, and Cav1.2 channels at PKA-dependent sites (Garcia-Alvarez et al 2015b). These findings suggest the existence of compensatory signaling mechanisms and complex functional interactions between STIM isoforms. The differences between single and double knockout models may arise from functional compensation, divergent roles of STIM1 and STIM2 in the PKA signaling pathway and AMPA receptor phosphorylation, as well as alterations in local calcium homeostasis. Together, these studies indicate that STIM2 is not only required for maintaining basal synaptic stability but also plays a critical role in activity-dependent synaptic plasticity. Future research should further investigate the mechanisms underlying STIM1-STIM2 interplay, which may clarify how these proteins exert complementary or opposing effects on memory formation and synaptic function.

#### STIM2 in presynaptic function and neurotransmitter release

In addition to its postsynaptic localization, STIM2 is abundantly localized in glutamatergic nerve terminals, where it is detected in ~ 60% of presynapses. Activation of the STIM2-dependent pathway in the hippocampal pyramidal neurons of mice and rats after short-term ER calcium depletion and increased calcium levels in presynaptic terminals enhance spontaneous vesicular glutamate release and regulate synaptic readiness for glutamatergic transmission without affecting GABA release. These effects are likely mediated through interaction with the presynaptic calcium sensor synaptotagmin-7 (Chanaday et al 2021). Interestingly, SOCE-dependent spontaneous glutamate release is physiologically inhibited by synaptotagmin-1. Under conditions of synaptotagmin-1 deficiency, STIM2 assumes a compensatory role, further enhancing presynaptic activity (Chanaday et al 2021). Conversely, inhibition of SOCE or synaptotagmin-7 reduces excessive neurotransmitter release and proapoptotic protein levels, and attenuates ER stress responses, linking STIM2 to neuroprotective mechanisms (Chanaday et al 2021).

#### Behavioral consequences of STIM2 dysregulation

At the behavioral level, studies using transgenic models have demonstrated that STIM2 and Orai1 overexpression produce only subtle effects on synaptic transmission and animal behavior, with effects primarily observed in females (Majewski et al 2020). No significant deficits in short-term plasticity or learning and memory were reported, suggesting that STIM2 functions as a fine modulator of calcium homeostasis and synaptic dynamics under physiological conditions rather than as a major driver of behavioral phenotypes.

Conversely, Berna-Erro et al. reported that STIM2 deficiency leads to specific cognitive deficits, particularly in hippocampus-dependent spatial learning and memory (Berna-Erro et al 2009). In the Morris water maze test, *Stim2*⁻/⁻ mice displayed normal motor performance but required more time and traveled longer distances to locate the hidden platform, indicating selective impairment of hippocampus-dependent learning processes.

These apparently divergent findings likely reflect differences in experimental models, genetic manipulation strategies, and the direction of STIM–Orai pathway modulation (Berna-Erro et al 2009, Majewski et al 2020). Additional support comes from double *Stim1/Stim2* knockout models, where the simultaneous loss of both proteins produces marked impairments in spatial learning and hippocampal-dependent memory, whereas single-gene deletion often results in relatively subtle phenotypes (Garcia-Alvarez et al 2015b). Interestingly, these behavioral deficits occur despite enhanced LTP, indicating that excessive or poorly controlled synaptic potentiation may itself impair neuronal network performance. Together, these findings suggest that effective cognitive processing depends on balanced regulation of synaptic plasticity rather than simply increased synaptic strengthening.

### Orai1

#### Localization and developmental regulation of Orai1

With respect to SOCE membrane channels, Orai1 has been functionally detected in the cell bodies of cortical neurons and in the neurites of hippocampal and cortical neurons (Gruszczynska-Biegala & Kuznicki 2013, Gruszczynska-Biegala et al 2011, Klejman et al 2009, Maneshi et al 2020, Secondo et al 2019). High-resolution imaging studies further revealed its presence in the heads of dendritic spines of hippocampal neurons, with the strongest expression observed at early developmental stages (approximately 1 week in culture) (Basnayake et al 2021, Korkotian et al 2017, Korkotian & Segal 2017, Maneshi et al 2020, Tshuva et al 2017). At later stages of maturation (approximately 3 weeks in culture), the proportion of spines containing Orai1 significantly decreases, suggesting developmental downregulation of Orai1 in dendritic compartments. It could be considered whether this reduction in Orai1 expression in maturing neurons reflects a shift from SOCE-dependent plasticity mechanisms toward more specialized forms of synaptic plasticity in mature neurons.

#### Orai1 in dendritic spines and ER calcium refilling

In dendritic spines, Orai1 contributes to local calcium signaling in cooperation with STIM proteins. Under conditions of reduced extracellular calcium, Orai1 colocalizes with STIM2 in dendritic spines, generating local calcium signals that activate downstream calcium-dependent cascades (Gruszczynska-Biegala & Kuznicki 2013, Korkotian et al 2017). Slow calcium influx through the STIM1–Orai1 pathway primarily supports replenishment of ER calcium stores within the spine apparatus (Korkotian et al 2014, Segal & Korkotian 2014). The close spatial association between SERCA pumps and Orai1-containing plasma membrane domains observed in spines containing the spine apparatus may facilitate efficient replenishment of ER calcium stores without triggering CICR, whereas the spine apparatus itself mediates CICR in response to activity-induced calcium signals (Basnayake et al 2021).

#### Orai 1 in presynaptic function and neurotransmitter release

Orai1 also contributes to presynaptic calcium regulation, particularly in cooperation with STIM2 (Chanaday et al 2021, Maciąg et al 2024). Localization of Orai channels near presynaptic calcium reservoirs enables rapid calcium store replenishment following synaptic activity, supporting precise synaptic transmission and spike fidelity. Through this mechanism, the STIM/Orai complex controls both the frequency and amplitude of vesicular neurotransmitter release, thereby influencing short- and long-term synaptic plasticity (Chanaday et al 2021). This modulation is particularly relevant for glutamatergic transmission and may depend on stimulation intensity and frequency. It would be interesting to determine whether Orai1 performs similar functions in other types of synapses, e.g., GABAergic synapses, and how its presynaptic activity interacts with mitochondrial calcium buffering mechanisms.

#### Orai1 in synaptic signaling, plasticity, and neuron–astrocyte communication

Maneshi and colleagues have shown that the role of Orai1 in the hippocampus is highly state-dependent rather than universal (Maneshi et al 2020). Deletion of this channel does not significantly affect basal synaptic transmission, presynaptic short-term plasticity, or dendritic spine density, whereas it is critically involved in activity-dependent calcium signaling triggered by glutamatergic receptor activation (Hori et al 2020, Maneshi et al 2020). Thus, Orai1 is dispensable for baseline synaptic function but becomes functionally engaged during synaptic stimulation.

Nevertheless, upon neuronal activation, Orai1 rapidly contributes to calcium influx into the cytoplasm and replenishment of ER stores. Its deletion selectively weakens glutamate receptor-evoked calcium signals, which does not result from nonspecific spine structural defects but rather directly from the loss of Orai1. This effect is particularly pronounced at physiological Mg^2^⁺ concentrations, where Orai1 enhances NMDA receptor-mediated calcium influx, effectively amplifying subthreshold postsynaptic signals (Maneshi et al 2020). Although increased NMDA receptor activation can only partially compensate for its loss, Orai1 remains a key regulator of calcium influx and ER homeostasis in dendritic spines. Accordingly, Orai1 may contribute to the amplification and maintenance of localized calcium signaling under conditions of weak synaptic stimulation. Future studies should examine whether a similar mechanism occurs in other brain regions.

Consistently, Orai1 deficiency in excitatory hippocampal neurons leads to impaired GluA1 receptor trafficking to the postsynaptic membrane, reduced CaMKII activation in dendritic spines, and diminished activity-dependent spine enlargement (Maneshi et al 2020). These molecular changes translate into reduced synaptically evoked calcium transients and deficits in LTP at Schaffer–CA1 synapses in hippocampal pyramidal neurons, which depend heavily on NMDA receptor-mediated calcium signaling. Together, these changes indicate that Orai1 is not required for basal synaptic transmission but is essential for the amplification and stabilization of activity-induced calcium signals underlying synaptic plasticity.

Beyond neuronal signaling, Orai1 also contributes to neuron–astrocyte communication within hippocampal networks. In mixed neuron-astrocyte cultures, LTP-like neuronal stimulation activates Orai1-mediated SOCE, engaging NMDA receptors in neurons and mGluR5 in astrocytes (Lim et al 2018). This pathway leads to calcineurin activation and its nuclear translocation in astrocytes, suggesting a role for Orai1 in bidirectional neuron–glia signaling and in the coordination of network-level plasticity and calcium homeostasis (Lim et al 2018). Overall, these findings support the view that Orai1 functions as a key amplifier of activity-dependent calcium signaling, integrating postsynaptic plasticity mechanisms with astrocytic modulation of neuronal networks. A remaining open question is whether disruption of Orai1-dependent signaling could alter astrocyte-mediated modulation of neuronal network plasticity and thereby influence memory-related processes.

#### Orai1 in structural plasticity and synaptic remodeling

Beyond its role in mature synapses, Orai1 also contributes to structural plasticity and synapse formation. In rat hippocampal neurons, knockdown of Orai1 or expression of a dominant-negative mutant disrupts key postsynaptic plasticity mechanisms. This leads to impaired dendritic spine maturation following chemically induced LTP, increased filopodia density, and reduced synaptic connectivity (Korkotian et al 2017, Korkotian & Segal 2017, Tshuva et al 2017). These findings suggest that Orai1-mediated calcium influx supports the transition of dendritic protrusions after contact with presynaptic terminals, into mature, fully functional spines (Korkotian et al 2017). Accordingly, Orai1 may contribute to early stages of synaptogenesis by providing localized calcium signals required for spine stabilization. Additionally, Orai1 expression declines in aged neurons, suggesting that reduced Orai1-dependent signaling may contribute to the loss of mature mushroom spines (Calvo-Rodríguez et al 2016).

Orai1 also links calcium signaling with metabolic and pathological processes. The relationship between the STIM1-Orai1 complex and the Niemann-Pick type C1 (NPC1) protein, which regulates cholesterol transport from lysosomes, highlights a functional connection between calcium homeostasis and lipid metabolism (Tiscione et al 2019). Disruption of NPC1 function, by NPC1 mutation or inhibition, leads to reduced ER calcium levels and compensatory upregulation of STIM1-Orai1-dependent SOCE, including increased expression of SOCE components. While short-term activation of this pathway may transiently increase dendritic spine formation, its chronic overactivation destabilizes synaptic structure, resulting in spine loss and impaired plasticity (Tiscione et al 2019). These observations represent a potential link between altered lipid metabolism, dysregulated SOCE, and synaptic dysfunction.

Similarly, mitochondrial dysfunction-induced elevations in intracellular calcium promote filopodia and dendritic spine formation in an Orai1-dependent manner (Kushnireva et al 2023). Application of the mitochondrial uncoupler carbonyl cyanide chlorophenylhydrazone (CCCP) induces these effects, which are abolished under calcium-free conditions, by the Orai1 antagonist 2-APB, or by expression of a dominant-negative Orai1 mutant (Kushnireva et al 2023). Together these findings support the view that Orai1-mediated calcium entry contributes to the initiation and stabilization of new synaptic structures under both physiological and stress-related conditions.

#### *Behavioral consequences and *in vivo* relevance of Orai1*

At the behavioral level, mice with *Orai1* deletion restricted to pyramidal neurons, while preserving its function in interneurons, show deficits in tasks assessing working and associative memory (Maneshi et al 2020). These findings indicate that Orai1-dependent calcium signaling in excitatory neurons contributes to hippocampus-dependent cognitive processes. Studies in transgenic mice overexpressing wild-type Orai1 in neurons (Tg(ORAI1)Ibd) further revealed pronounced sex- and age-dependent effects (Maciąg et al 2019). In females, Orai1 overexpression in neurons leads to synaptic transmission disturbances and cognitive deficits, manifested by reduced hippocampal synaptic activity, impaired context recognition, and spontaneous seizure-like episodes in older individuals. In contrast, males display reduced anxiety-like behavior without significant alterations in synaptic transmission or memory performance (Maciąg et al 2019). These observations suggest that the functional impact of Orai1 dysregulation may depend on sex-specific regulatory mechanisms. The observed sex differences further raise the possibility that Orai1 function may be modulated by steroid hormones or gene regulatory pathways in a sex-dependent manner, although this requires further investigation.

In addition to its role in hippocampal circuits, Orai1-mediated SOCE also regulates neuronal excitability through modulation of Kv4-containing A-type voltage-gated potassium channels in the mouse spinal cord, thereby influencing nociceptive processing and learning-related behaviors (Dou et al 2018). Together, these findings indicate that Orai1 contributes to behavior by integrating calcium-dependent signaling across multiple neural circuits.

Evidence from invertebrate models further supports the behavioral relevance of Orai-dependent calcium signaling. In Drosophila, neuronal knockdown of dOrai (the homolog of mammalian Orai) or dSTIM leads to severe defects in flight behavior, associated with impaired rhythmic firing of motor circuits (Venkiteswaran & Hasan 2009). These findings indicate a key role of SOCE in maintaining calcium homeostasis and central pattern generator activity, supporting a conserved function of Orai-mediated calcium entry in motor behavior.

Collectively, these observations establish Orai1-dependent SOCE as a fundamental regulator of neuronal performance across species, linking intracellular calcium dynamics to both cognitive and motor aspects of behavior.

### Orai2 and Orai3

#### Orai2 in dendritic spines and SOCE signaling

Orai2 appears to play a prominent role in regulating calcium dynamics in dendritic spines, particularly in hippocampal neurons, although its exact spatial distribution remains somewhat variable across studies. Some reports indicate relatively higher enrichment in dendritic spines compared to Orai1, suggesting a stronger contribution to local calcium regulation under basal conditions (Courjaret et al 2024, Zhang et al 2016, Zhang & Hu 2020), whereas other observations describe a more diffuse distribution within dendritic shafts and lower enrichment within spines (Korkotian et al 2017).

Interestingly, imaging studies demonstrated that acute extracellular calcium removal does not induce major redistribution of either Orai1 or Orai2 between dendritic shafts and spines (Korkotian et al 2017). However, under calcium-free conditions, Orai1 exhibited significantly increased colocalization with STIM2, whereas a similar enhancement was not observed for Orai2. Moreover, mushroom spines displayed a reduction in STIM2/Orai2 puncta below control levels following calcium withdrawal (Korkotian et al 2017). These observations suggest that Orai2-containing complexes may be less dynamically recruited during acute calcium depletion and instead preferentially support constitutive or homeostatic calcium signaling under resting conditions. In contrast, Orai1-associated complexes appear to respond more robustly to rapid fluctuations in extracellular calcium availability.

Functionally, Orai2 operates in close cooperation with STIM2, supporting constitutive low-level calcium entry required for spine stability and homeostatic synaptic regulation (Zhang et al 2016). In contrast to classical SOCE triggered by pronounced ER calcium release, STIM2–Orai2 signaling appears to preferentially regulate local basal cytosolic calcium levels and maintain calcium homeostasis under resting conditions. Although Orai2 also contributes to mGluR-dependent intracellular calcium signaling (Chen-Engerer et al 2019), its function appears to be more closely associated with fine-tuning local calcium dynamics than with large-scale ER calcium depletion responses. Mechanistically, Orai2 has been implicated in IP_3_R-dependent calcium signaling and ER calcium replenishment without significantly affecting RyR-controlled calcium dynamics, similarly to STIM1-associated pathways (Chen-Engerer et al 2019, Nomura et al 2025). This suggests functional specialization of distinct ER calcium pools and signaling routes.

Under resting conditions, partial depletion of ER calcium stores can activate STIM1 protein and induce its clustering near the plasma membrane, where it colocalizes with both Orai1 and Orai2 (Lalonde et al 2014). Although the specific channel mediating basal SOCE in this context has not been directly identified, Orai2 has been proposed as a likely contributor to this low-level calcium entry. This basal SOCE pathway has further been linked to transcriptional regulation, including activity-dependent control of Sp4, suggesting a coupling between Orai2-associated calcium signaling, homeostatic plasticity, and gene expression programs (Lalonde et al 2014).

#### Orai2 in synaptic plasticity and spine stability

Studies by Zhang et al. showed that, at the synaptic level, Orai2 forms a functional complex with TRPC6 and STIM2, which mediates SOCE in mature mushroom-type dendritic spines of the mouse hippocampal neurons (Zhang et al 2016). While STIM2 interactions were detected with both Orai1 and Orai2, selective reduction of Orai2, but not Orai1, leads to a loss of mature spines and a significant decrease in spine SOCE activity. Furthermore, Orai2 knockdown resulted in reduced PSD95 expression and decreased pCaMKII levels, indicating that Orai2-dependent calcium signaling supports stabilization and maintenance of postsynaptic signaling competence in mature hippocampal neurons. In contrast, Orai1 may contribute more prominently in activity-dependent calcium dynamics or earlier stages of dendritic development (Zhang et al 2016).

In striatal MNSs, Orai1 and Orai2 form functional SOCE complexes together with STIM1 and STIM2 proteins (Wu et al 2018, Wu et al 2016). Synaptosomal analyses from wild-type mouse striata revealed preferential interactions of STIM1 and STIM2 with Orai1 and Orai2, supporting their involvement in local calcium signaling within dendritic spines. Consistently, RNAi-mediated silencing of either Orai1 or Orai2 reduced spine SOC activity, confirming their contribution to calcium entry in MSN spines. However, diminished SOC caused by individual knockdown of Orai1 or Orai2 did not translate into significant changes in dendritic spine density (Wu et al 2018). This indicates that, unlike in hippocampal neurons where Orai2 is crucial for mature spine maintenance (Zhang et al 2016), MSN spine stability may rely more heavily on upstream ER calcium sensing mechanisms mediated by STIM proteins, potentially due to compensatory interactions among multiple SOC channel components.

#### Behavioral relevance of Orai2

Despite its clear role at the synaptic level, Orai2 appears to have a relatively modest impact on behavior. Orai2 knockout mice do not show significant impairments in spatial memory or learning in Barnes maze tests (Stegner et al 2019). However, subtle alterations observed in exploratory and avoidance behaviors including reduced climbing and less frequent entry to the center of the open field, may indicate a subtle influence of Orai2 on fine-tuning of neuronal network function (Stegner et al 2019). These observations raise the possibility that Orai2-dependent calcium signaling participates in the regulation of affective or adaptive behavioral responses, although its contribution appears less pronounced and more context-dependent than that of Orai1. A question arises as to whether these subtle behavioral changes might be significant for modulation of hippocampal network excitability or broader circuit-level regulation (Maciąg et al 2019).

#### Orai3 in neuronal calcium signaling

In contrast to Orai1 and Orai2, relatively little is known about the role of Orai3 in central neurons, especially at synapses. It has been established that Orai3, like Orai1, is an important component of SOCE channels in DRG neurons. Calcium influx through these channels increases membrane depolarization and neuronal excitability, indirectly affecting synaptic activity. Functional studies indicate that Orai3 knockdown partially reduces SOCE, whereas simultaneous silencing of Orai1 and Orai3 nearly abolishes calcium influx, suggesting that both proteins may form functional homomultimers (Wei et al 2017). In contrast, Orai2 knockdown has a less pronounced effect in this system (Wei et al 2017, Xia et al 2014). However, this does not exclude involvement of Orai2 in SOCE at synaptic sites, as this contribution has not been directly assessed. These findings suggest that Orai3 may play a more prominent role in peripheral sensory neurons such as DRG neurons or specialized cell types, whereas its contribution to synaptic calcium signaling in central neurons remains unclear. Further studies are required to determine whether Orai3 participates in dendritic spine SOCE or is restricted to non-synaptic neuronal populations and glial cells.

Additional evidence supporting a limited role of Orai3 in central synaptic regulation comes from studies in striatal MSNs, where Orai3 knockdown did not affect dendritic spine density under physiological conditions (Wu et al 2018). Moreover, unlike silencing of Orai1 or Orai2, reduction of Orai3 failed to rescue spine loss in YAC128 MSNs, a cellular model of Huntington’s disease-associated synaptic degeneration. These findings suggest that Orai3 is not a major contributor to neuronal SOC pathways involved in dendritic spine maintenance or pathological spine remodeling in the striatum. Together, these observations further support the notion that Orai3 may play a more specialized or context-dependent role in neuronal calcium signaling compared with the more prominent synaptic functions of Orai1 and Orai2.

## Summary and perspectives

**Fig. 1:**
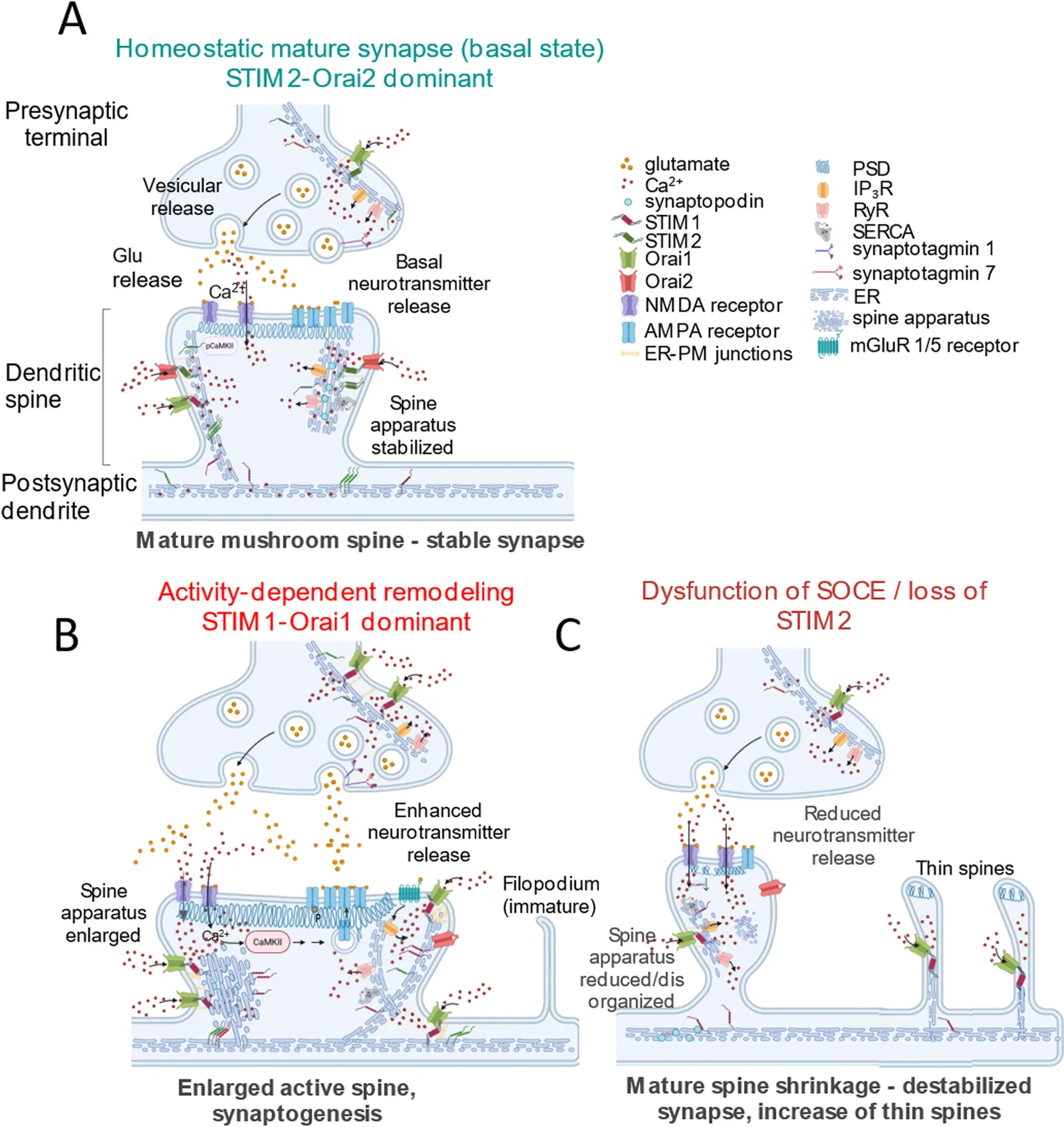
Current model of isoform-specific STIM and Orai signaling in hippocampal synapses and dendritic spines. STIM proteins are depicted within the ER membrane, whereas Orai channels are localized to the plasma membrane. Arrows indicate direction of calcium influx and neurotransmitter release. Image was created with BioRender.com. **A** Homeostatic mature synapse under basal conditions dominated by STIM2–Orai2 signaling. STIM2 is enriched in mature mushroom spines, where it cooperates primarily with Orai2 to sustain low-level basal SOCE and maintain calcium homeostasis. This signaling supports stabilization of the spine apparatus, maintenance of PSD95/pCaMKII signaling, receptor composition, and mature spine architecture. Basal neurotransmitter release and tonic glutamatergic signaling are preserved. **B** Activity-dependent synaptic remodeling dominated by STIM1–Orai1 signaling. Strong synaptic activity, glutamate release, and ER Ca^2^⁺ depletion promote STIM1 oligomerization and recruitment to ER–PM junctions, where STIM1 activates Orai1-mediated SOCE. This pathway amplifies local calcium signaling near NMDA receptors and mGluR1/5 receptors, enhances presynaptic neurotransmitter release, and promotes rapid ER remodeling, spine enlargement, filopodia formation, and AMPAR recruitment. These processes drive activity-dependent structural plasticity associated with late-phase LTP and synaptogenesis. **C** Loss of STIM2 reduces basal SOCE and impairs Ca^2^⁺ homeostasis. Reduced STIM2-dependent SOCE decreases pCaMKII/PSD95 signaling and disrupts spine apparatus organization. These alterations lead to shrinkage of mature mushroom spines, destabilization of synaptic structure, impaired receptor maintenance, and an increased proportion of immature, thin and unstable spines. Presynaptic Ca^2^⁺ signaling and neurotransmitter release are reduced, while NMDAR-mediated Ca^2^⁺ influx persists, contributing to dysfunctional synaptic signaling and impaired synaptic stability

STIM1 and STIM2 proteins together with Orai channels form a highly specialized calcium signaling network that enables neurons to maintain intracellular calcium homeostasis while dynamically regulating synaptic strength and structural plasticity. Although initially described in non-excitable cells, the neuronal STIM/Orai pathways are now recognized as key regulators of dendritic spine organization, ER–PM communication, synapse stability, and multiple forms of synaptic plasticity. As summarized in Table 1 and Fig. 1, current evidence indicates that neuronal SOCE proteins operate through highly compartmentalized and activity-dependent mechanisms whose functions differ substantially across neuronal types, developmental stages, and experimental conditions.

This overview further highlights that many key functional insights into neuronal SOCE proteins have emerged from genetic loss-of-function and in vivo models, particularly under conditions requiring activity-dependent synaptic plasticity rather than during basal neuronal transmission. Importantly, findings obtained using knockout (KO), conditional knockout (cKO), knockdown (KD), and overexpression systems (OE) frequently reveal context-dependent and sometimes region-specific effects, emphasizing the complexity of STIM/Orai signaling in neurons.

Among STIM proteins, STIM1 and STIM2 represent complementary yet partially overlapping regulators of neuronal calcium signaling whose functions differ in activation threshold, spatial localization, and contribution to synaptic plasticity. STIM1 is preferentially recruited during strong synaptic activity and pronounced ER calcium depletion, where it promotes dynamic ER–PM remodeling, transient SOCE activation, and activity-dependent structural plasticity of dendritic spines (Fig. 1B). In contrast, STIM2 is constitutively enriched in mature mushroom-type spines as well as at perisynaptic ER–PM junctions and responds to subtle fluctuations in ER calcium levels, thereby sustaining constitutive low-level SOCE and supporting basal calcium homeostasis in mature synapses (Fig. 1A). This functional distinction is reflected in their molecular interactions. STIM1 predominantly participates in stimulus-evoked signaling complexes associated with rapid synaptic remodeling, whereas STIM2 is more tightly linked to cytoskeletal scaffolds, synaptopodin, and receptor-associated signaling complexes that stabilize ER positioning, receptor composition, and spine architecture. Consequently, STIM1 is more strongly associated with dynamic and transient forms of synaptic regulation, including mGluR-dependent LTD, presynaptic remodeling, filopodia formation, and activity-dependent spine plasticity. In contrast, STIM2 primarily maintains stable calcium microdomains required for long-term structural and functional plasticity, receptor homeostasis, and neuronal survival. This divergence may explain why reduced STIM2 levels are consistently associated with destabilization of mature mushroom spines, spine apparatus disorganization, impaired receptor trafficking, and long-term synaptic deficits (Fig. 1C), whereas STIM1 dysfunction more prominently affects adaptive or stimulus-dependent plasticity mechanisms.

At the postsynaptic level, STIM1 primarily supports transient, activity-dependent calcium signaling, whereas STIM2 stabilizes basal calcium levels and maintains glutamatergic receptor function, suggesting that the balance between these proteins is crucial for preserving optimal excitability and network plasticity. The relative contribution of each STIM isoform is likely shaped by neuronal activity patterns, ER calcium store dynamics, and receptor-specific signaling pathways, although the molecular mechanisms governing transitions between STIM1-dominated and STIM2-dominated signaling modes remain incompletely understood.

Evidence from genetic models further supports this functional specialization. Single-gene deletion of *Stim1* or *Stim2* frequently produces selective or relatively subtle phenotypes under basal conditions (Berna-Erro et al 2009, Garcia-Alvarez et al 2015b, Ryu et al 2017), suggesting partial functional compensation between isoforms at the level of overall neuronal calcium homeostasis. However, conditional genetic deletion of *Stim2* in hippocampal neurons demonstrated that STIM1 cannot fully compensate for the specific role of STIM2 in maintaining synaptic SOCE and mature mushroom spine stability (Sun et al 2014). Consistently, combined *Stim1/Stim2* deletion results in enhanced LTP despite impaired hippocampal-dependent memory, demonstrating that coordinated STIM signaling is required for normal cognitive function. These findings further suggest that STIM-dependent pathways not only support synaptic plasticity but also constrain excessive potentiation to preserve network stability and proper information processing (Garcia-Alvarez et al 2015b).

Similar functional specialization is observed among Orai isoforms. Orai1 is strongly associated with rapid, activity-dependent SOCE and dynamic synaptic remodeling. In dendritic spines, Orai1 amplifies NMDA receptor-mediated calcium signaling, supports ER calcium refilling, regulates GluA1 trafficking, and contributes to activity-dependent spine enlargement and synaptogenesis (Fig. 1B). Orai1 additionally participates in presynaptic neurotransmitter release and neuron–astrocyte communication, emphasizing its multidimensional role in neuronal network regulation. Conditional deletion of *Orai1* in pyramidal neurons does not substantially affect basal synaptic transmission or spine density but markedly impairs glutamatergic calcium signaling, LTP induction, and memory-related behaviors, indicating that Orai1 becomes critically engaged during periods of elevated synaptic demand rather than under resting conditions (Maneshi et al 2020). Conversely, neuronal overexpression of Orai1 produces sex- and age-dependent phenotypes, including altered synaptic transmission, cognitive deficits, seizure-like activity, and behavioral alterations (Maciąg et al 2019). Together, these findings demonstrate that both insufficient and excessive Orai1 activity can destabilize neuronal network function, highlighting the importance of tightly regulated SOCE dynamics.

In contrast, Orai2 appears to function predominantly within constitutive or low-level SOCE pathways associated with maintenance of mature dendritic spines and homeostatic signaling (Fig. 1A). Orai2, particularly in cooperation with STIM2, supports sustained low-level calcium influx required for the maintenance of PSD95/CaMKII signaling and postsynaptic signaling competence in mature hippocampal neurons. Although Orai2 contributes to spine SOCE in multiple neuronal populations, its role in structural spine maintenance appears region-specific. In hippocampal neurons, reduction of Orai2 destabilizes mature spines and impairs homeostatic signaling, whereas in striatal medium spiny neurons reduced Orai2-dependent SOCE does not significantly alter spine density, suggesting compensatory interactions among multiple SOCE components. Consistent with these findings, Orai2 knockout mice display relatively subtle and context-dependent behavioral phenotypes compared with Orai1 models (Stegner et al 2019).

Compared with Orai1 and Orai2, the neuronal role of Orai3 remains poorly understood. Current evidence is derived primarily from DRG neurons and striatal MSN models, where Orai3 contributes to SOCE and neuronal excitability, often together with Orai1. However, Orai3 does not appear to play a major role in dendritic spine maintenance or synaptic structural remodeling under physiological conditions, suggesting a more specialized or context-dependent function.

Collectively, current evidence supports the concept that neuronal SOCE involves functionally distinct but partially overlapping STIM–Orai signaling pathways. The STIM1–Orai1 pathway is primarily associated with rapid, activity-dependent calcium entry and transient synaptic remodeling whereas the STIM2–Orai2 pathway contributes more prominently to basal calcium homeostasis and long-term synaptic stability (Fig. 1). However, increasing evidence suggests that these pathways do not operate fully independently. Instead, they may act cooperatively and dynamically interact within shared ER–PM signaling microdomains. For example, activity-dependent STIM1–Orai1 signaling during late-phase LTP may facilitate recruitment or stabilization of STIM2–Orai2 complexes at ER–PM junctions. Future studies will be required to better define how these signaling pathways intersect and coordinate neuronal calcium homeostasis and synaptic plasticity, particularly across different neuronal populations and neuron-glia interactions. It also remains an open question whether differences in STIM/Orai dynamics contribute to age-dependent pathological alterations in synaptic plasticity. Disruption of either signaling axis destabilizes calcium homeostasis and impairs synaptic function, highlighting the importance of coordinated STIM–Orai signaling for long-term neuronal stability and information processing.

At the same time, the available data emphasize substantial heterogeneity in neuronal SOCE mechanisms across brain regions, developmental stages, and cell types. Differences observed between KO, cKO, KD, and OE models indicate that STIM/Orai proteins operate within tightly balanced signaling networks in which both reduced and excessive activity may impair neuronal performance. Moreover, several important questions remain unresolved, including how STIM1/STIM2 switching is regulated at active synapses, how Orai subtype composition changes across synapse types during development and aging, and how neuronal SOCE interacts with astrocytic signaling and mitochondrial calcium buffering systems. Future studies should also determine whether selective modulation of STIM/Orai signaling pathways could provide therapeutic benefit in neurodegenerative and neuropsychiatric disorders characterized by impaired synaptic plasticity and memory dysfunction.

Understanding how STIM and Orai proteins orchestrate ER architecture, receptor trafficking, dendritic spine morphology, and synaptic transmission efficiency provides important insights into the molecular basis of learning and memory. This is particularly relevant, as accumulating evidence suggests that these proteins may exert neuroprotective functions by preserving calcium homeostasis and synaptic integrity in disorders such as Alzheimer’s disease, Huntington’s disease, epilepsy, and ischemic injury (Berna-Erro et al 2009, Bojarski et al 2009, Deng et al 2020, Hori et al 2020, Secondo et al 2019, Stegner et al 2019, Steinbeck et al 2011, Sun et al 2014, Tong et al 2016, Wu et al 2018, Wu et al 2016, Wu et al 2011, Zhang et al 2016, Zhang et al 2015).

## Data Availability

No datasets were generated or analysed during the current study.
